# Genetic and epigenetic determinants of diffuse large B-cell lymphoma

**DOI:** 10.1038/s41408-020-00389-w

**Published:** 2020-12-04

**Authors:** Tanner J. Bakhshi, Philippe T. Georgel

**Affiliations:** 1 Joan C. Edwards School of Medicine, Marshall University, Huntington, WV 25755 USA; 2 Department of Biological Sciences, Cell Differentiation and Development Center, Byrd Biotechnology Science Center, Marshall University, Huntington, WV 25755 USA

**Keywords:** Diseases, Health care

## Abstract

Diffuse large B-cell lymphoma (DLBCL) is the most common type of lymphoma and is notorious for its heterogeneity, aggressive nature, and the frequent development of resistance and/or relapse after treatment with standard chemotherapy. To address these problems, a strong emphasis has been placed on researching the molecular origins and mechanisms of DLBCL to develop effective treatments. One of the major insights produced by such research is that DLBCL almost always stems from genetic damage that occurs during the germinal center (GC) reaction, which is required for the production of high-affinity antibodies. Indeed, there is significant overlap between the mechanisms that govern the GC reaction and those that drive the progression of DLBCL. A second important insight is that some of the most frequent genetic mutations that occur in DLBCL are those related to chromatin and epigenetics, especially those related to proteins that “write” histone post-translational modifications (PTMs). Mutation or deletion of these epigenetic writers often renders cells unable to epigenetically “switch on” critical gene sets that are required to exit the GC reaction, differentiate, repair DNA, and other essential cellular functions. Failure to activate these genes locks cells into a genotoxic state that is conducive to oncogenesis and/or relapse.

## Clinical aspects of diffuse large B-cell lymphoma

### Definition and epidemiology

Diffuse large B-cell lymphoma (DLBCL) is a hematological malignancy derived from mature B-cells that have undergone (or continue to undergo) the germinal center (GC) reaction in response to antigen and Helper T-cell stimulation. The name “DLBCL” stems from the fact that it consists of large, neoplastic B-cells that are diffusely spread throughout lymph nodes and, in some cases, extranodal tissues. The designation of DLBCL as a lymphoma means that it arises from lymphoid rather myeloid cells and is a solid rather than a “liquid” malignancy (e.g., leukemia). Specifically, DLBCL is classified as a type of Non-Hodgkin’s lymphoma (NHL).

For context, in 2019, NHL is estimated to be the seventh-most common type of cancer in the U.S., with an estimated 74,200 new cases that represent ~4.2% of all new cancer cases (SEER 1, https://seer.cancer.gov/statfacts/html/all.html). Data recorded between 2012 and 2016 in the U.S. show that NHL has an incidence rate of 19.6 per 100,000 persons per year and, in 2016, had an estimated prevalence of 694,704 patients (SEER 2, https://seer.cancer.gov/statfacts/html/nhl.html). Specifically, DLBCL is the most common subtype of NHL, accounting for 25–30% of NHL cases in the U.S.^1–3^. (UpToDate 1, https://www.uptodate.com/contents/epidemiology-clinical-manifestations-pathologic-features-and-diagnosis-of-diffuse-large-b-cell-lymphoma), and is also the most common type of lymphoma overall^1^ (UpToDate 1, https://www.uptodate.com/contents/epidemiology-clinical-manifestations-pathologic-features-and-diagnosis-of-diffuse-large-b-cell-lymphoma). Based on the same 2012–2016 U.S. dataset, DLBCL has an incidence rate of 5.6 per 100,000 persons per year overall and is more common in males (6.7 per 100,000 persons) than in females (4.6 per 100,000 persons) (SEER 3, https://seer.cancer.gov/statfacts/html/dlbcl.html). While there is no consensus on what causes the discrepancy between the incidence rates of DLBCL in males and females, there is evidence suggesting that differences in sex hormones may be partially responsible. Results from multiple studies indicate that pregnancy, live birth, and oral contraceptives are all associated with a reduced risk of DLBCL in females. The mechanism by which these effects are achieved is also unclear, although the direct and indirect effects of estrogen on multiple types of immune cells have been proposed^4^. DLBCL can occur in people of all ages, but cases are not evenly distributed amongst different age groups. The median age of diagnosis is 66 years old, with 25.0% of cases occurring between ages 65 and 74, 21.2% of cases between ages 55 and 64, and 20.1% cases between ages 75 and 84. The incidence rates of DLBCL in all other age groups are lower (e.g., 12.3% of cases between ages 45 and 54 and 8.8% of cases over the age of 84) (SEER 3, https://seer.cancer.gov/statfacts/html/dlbcl.html). DLBCL is also more common in Hispanics (i.e., Latinos) and Whites than in Asian/Pacific Islanders, Blacks (i.e., African Americans), and Native Americans/Alaskan Natives (SEER 3, https://seer.cancer.gov/statfacts/html/dlbcl.html). In addition to differences in incidence, ethnicity can also sometimes be associated with differences in clinical outcome. For instance, African-American DLBCL patients tend to be younger (mean age 54), are more likely to present at an advanced stage, and have lower survival and higher mortality rates^5^ (UpToDate 1, https://www.uptodate.com/contents/epidemiology-clinical-manifestations-pathologic-features-and-diagnosis-of-diffuse-large-b-cell-lymphoma). Because epidemiological data for DLBCL (i.e., not the broader classification of NHL) at the global level are scarce^6^, the data presented here are limited to the United States. The most comprehensive epidemiological database available for DLBCL is the United States’ National Cancer Institute (NCI) Surveillance, Epidemiology, and End Results (SEER) Program.

### Clinical presentation and diagnostic workup

**Fig. 1:**
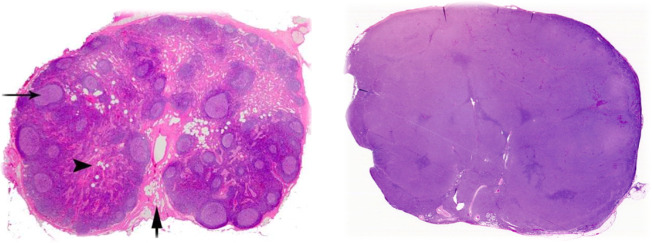
Comparison of normal and DLBCL-infiltrated lymph node histology. (Left) Normal lymph node after hematoxylin and eosin (H&E) staining. Note the complex and varied architecture. Arrow on the left points to a germinal center within a follicle; both are in the cortex (outer region). Arrowhead indicates the medulla (inner region). Bottom arrow shows the hilum, where blood and efferent lymph vessels are connected (Image Source: https://www.pathpedia.com/education/eatlas/histology/lymph_node/images.aspx?6 (Slide 1)). (Right) H&E staining of a lymph node that has been infiltrated by DLBCL. Note the glassy, uniform surface and complete loss of normal structures (Image Source: https://www.webpathology.com/image.asp?case=822&n=3 (Slide 3)).

Clinically, DLBCL often presents as a fast-growing symptomatic mass in the neck or abdomen, which is typically indicative of an enlarged lymph node. It most commonly occurs as an isolated event (de novo), but it can also transform from pre-existing lymphoid malignancies, such as follicular lymphoma (FL) and chronic lymphocytic leukemia (CLL). About 30% of patients also present with constitutional, or “B” symptoms (e.g., unexplained weight loss, fever, and night sweats), and over 50% of patients show an increase in their serum level of lactate dehydrogenase (LDH)^7,8^ (UpToDate 1, https://www.uptodate.com/contents/epidemiology-clinical-manifestations-pathologic-features-and-diagnosis-of-diffuse-large-b-cell-lymphoma). The typical evaluation of a patient exhibiting symptoms of NHL includes a complete blood count (CBC) with differential, a comprehensive metabolic panel (CMP) (including LDH and uric acid), tests for hepatitis B and human immunodeficiency virus (HIV), the determination of cardiac ejection fraction, a positron emission tomography with computed tomography (PET/CT) scan, a bone marrow biopsy (depending on PET/CT results), and an excisional lymph node biopsy (UpToDate 2, https://www.uptodate.com/contents/evaluation-staging-and-response-assessment-of-non-hodgkin-lymphoma) (UpToDate 3, https://www.uptodate.com/contents/clinical-presentation-and-diagnosis-of-non-hodgkin-lymphoma). The lymph node biopsy is crucial, because morphological analysis and immunophenotyping of affected tissue(s) are required for accurate diagnosis of DLBCL (UpToDate 1, https://www.uptodate.com/contents/epidemiology-clinical-manifestations-pathologic-features-and-diagnosis-of-diffuse-large-b-cell-lymphoma). Under the microscope, a lymph node that has been infiltrated by DLBCL usually exhibits a complete loss of normal structures and compartments (e.g., cortex, medulla, and follicles) and instead consists of diffuse sheets of neoplastic B-cells (Fig. 1). Though extensive morphological variation exists, the cells usually appear large and atypical, with enlarged nuclei, prominent nucleoli, and have a proliferation fraction (Ki67+) of over 40%. Immunohistochemistry (IHC) or flow cytometry should also show that the cells express standard B-cell markers, including cluster of differentiation (CD)19, CD20, CD22, CD45, and CD79a. Surface or cytoplasmic immunoglobulin (usually immunoglobulin M (IgM)) is expressed in 50–75% of DLBCL tumors as well^2,3^ (UpToDate 1, https://www.uptodate.com/contents/epidemiology-clinical-manifestations-pathologic-features-and-diagnosis-of-diffuse-large-b-cell-lymphoma).

### Staging and subtyping

The results of a patient’s PET/CT scan are used to stage his/her DLBCL. Since DLBCL is a type of NHL, staging is conducted according to the Lugano classification (Table 1), which goes by the number and location of tumor sites^9,10^ (UpToDate 2, https://www.uptodate.com/contents/evaluation-staging-and-response-assessment-of-non-hodgkin-lymphoma). Stage I involves one lymph node region or one extralymphatic site without lymph node involvement (Stage IE). Stage II involves two or more lymph node regions on the same side of the diaphragm, either with (Stage IIE) or without the localized involvement of an extralymphatic site. Together, Stages I and II constitute limited-stage disease. Stage III involves lymph nodes on both sides of the diaphragm. Stage IV requires the diffuse involvement of one or more extralymphatic organ(s), with or without the involvement of lymph nodes. Together, Stages III and IV constitute advanced-stage disease^9,10^. (UpToDate 2, https://www.uptodate.com/contents/evaluation-staging-and-response-assessment-of-non-hodgkin-lymphoma). The Lugano classification sometimes lacks clinical utility, in part because the staging system from which it was derived (Ann Arbor) was initially designed for Hodgkin’s lymphoma (HL). Unlike HL, NHL tends to spread more through the blood than through the lymphatic system, and most patients diagnosed with aggressive NHL are already at Stage III or IV by the time that they present with symptoms (50–70% of DLBCL patients, depending on the reference)^2,3,7,8,11,12^ (UpToDate 1, https://www.uptodate.com/contents/epidemiology-clinical-manifestations-pathologic-features-and-diagnosis-of-diffuse-large-b-cell-lymphoma).

**Table 1:** The Lugano classification.

Stage	Involvement	Extranodal status
Limited		
I	One node or a group of adjacent nodes	Single extranodal lesion with no nodal involvement
II	Two or more nodal groups on the same side of the diaphragm	Stage I or II: nodal extent with limited contiguous extranodal involvement
II Bulky	Like Stage II, but with “bulky” disease	N.A.
Advanced		
III	Nodes on both sides of the diaphragm; nodes above the diaphragm (spleen)	N.A.
IV	Additional non-contiguous extra-lymphatic involvement	N.A.

This is the system that is used to stage NHLs, including DLBCL, based on PET/CT scan results. Clinical outcomes can be quite different for patients with limited-stage vs. advanced-stage DLBCL (Table based on Table Source: UpToDate 2, https://www.uptodate.com/contents/evaluation-staging-and-response-assessment-of-non-hodgkin-lymphoma (Table 9) and Cheson et al.^10^).

It is now understood that DLBCL also encompasses a variety of subtypes that are morphologically indistinguishable yet exhibit distinct gene expression profiles and patterns of genetic and epigenetic aberrations. Though multiple subtyping schemes have been developed^13–16^, only the original system published by Alizadeh et al.^17^ has been officially adopted by the World Health Organization (WHO)^2,3^. This system uses information gathered from gene expression profiling (GEP)^17^, IHC algorithms^18,19^, or the Lymph2Cx gene expression assay^20^ to classify DLBCL into two main subtypes based on its probable cell of origin (COO): germinal center B-cell (GCB) and activated B-cell (ABC) (UpToDate 4, https://www.uptodate.com/contents/prognosis-of-diffuse-large-b-cell-lymphoma). A third, minor subtype consists of cases that cannot be classified as either GCB or ABC. The GCB subtype accounts for ~40% of de novo DLBCL cases, while the ABC subtype and other non-GCB DLBCLs account for the other ~60% of de novo cases^21–23^ (UpToDate 5, https://www.uptodate.com/contents/initial-treatment-of-advanced-stage-diffuse-large-b-cell-lymphoma). Clinically, in addition to COO classification, DLBCL is also further stratified by the presence of *BCL2* (B-cell CLL/lymphoma 2), *BCL6* (B-cell CLL/lymphoma 6), and *MYC* (Myelocytomatosis) chromosomal translocations and/or expression, as determined by fluorescent in situ hybridization (FISH) or IHC, respectively.

### Standard treatment and clinical outcomes

The standard treatment for DLBCL is the R-CHOP chemoimmunotherapy regimen (Rituximab, Cyclophosphamide, Hydroxydaunorubicin (doxorubicin), Oncovin (vincristine), and Prednisone). Rituximab is a monoclonal antibody that binds the CD20 protein on the surface of B-cells and triggers an innate immune reaction, leading to cellular toxicity (UpToDate 6, https://www.uptodate.com/contents/rituximab-intravenous-including-biosimilars-of-rituximab-drug-information). Cyclophosphamide is an alkylating agent that cross-links the strands of DNA and inhibits DNA replication (UpToDate 7, https://www.uptodate.com/contents/cyclophosphamide-drug-information). Doxorubicin is an intercalating agent that binds between DNA base pairs and inhibits DNA replication, DNA repair, and transcription (UpToDate 8, https://www.uptodate.com/contents/doxorubicin-conventional-drug-information). Vincristine is a tubulin-binding agent that inhibits the formation of microtubules and the mitotic spindle, which prevents the completion of mitosis (UpToDate 9, https://www.uptodate.com/contents/vincristine-conventional-drug-information). And, prednisone is a corticosteroid (glucocorticoid) that acts as an immunosuppressant and anti-inflammatory agent (UpToDate 10, https://www.uptodate.com/contents/prednisone-drug-information). Historically, the CHOP regimen (even before the addition of rituximab) has been the treatment of choice for DLBCL based on its performance in clinical trials^24–26^. Other regimens to which CHOP was compared failed to demonstrate an increase in overall survival (OS), disease-free survival (DFS), or remission rate (RR), and some [e.g., m-BACOD (methotrexate with leucovorin, bleomycin, cyclophosphamide, vincristine, and dexamethasone) and MACOP-B (methotrexate with leucovorin, doxorubicin, cyclophosphamide, vincristine, prednisone, and bleomycin)^27^] were associated with an increase in toxicity^27–32^ (UpToDate 5, https://www.uptodate.com/contents/initial-treatment-of-advanced-stage-diffuse-large-b-cell-lymphoma).

The specifics of R-CHOP therapy, as well as the extent to which patients respond, vary depending on the stage and/or molecular subtype of DLBCL. For cases of limited-stage DLBCL (30–40% of patients) (UpToDate 11, https://www.uptodate.com/contents/initial-treatment-of-limited-stage-diffuse-large-b-cell-lymphoma), molecular subtype can still be clinically relevant, but it does not guide decisions related to treatment as much as it does for advanced-stage DLBCL (UpToDate 11, https://www.uptodate.com/contents/initial-treatment-of-limited-stage-diffuse-large-b-cell-lymphoma). Instead, an important decision regarding the treatment of limited-stage DLBCL is whether to use R-CHOP alone or in combination with involved-field radiation therapy (IFRT). National Comprehensive Cancer Network guidelines published in 2010 (ref. ^33^) recommended treating limited-stage DLBCL with either three cycles of R-CHOP and subsequent IFRT or six to eight cycles of R-CHOP (with or without subsequent IFRT). This has been heavily debated due to concerns over potentially unnecessary radiation-induced toxicity. Administration of the former (3 cycles R-CHOP + IFRT) has been associated with a 5-year OS rate of ~95% (though individual patient outcomes can vary)^34^, as well as lower acute hematologic and cardiac toxicity (UpToDate 11, https://www.uptodate.com/contents/initial-treatment-of-limited-stage-diffuse-large-b-cell-lymphoma). However, the latter (6–8 cycles R-CHOP − IFRT) is associated with a comparable long-term survival rate and avoids the risk of long-term radiation toxicity (UpToDate 11, https://www.uptodate.com/contents/initial-treatment-of-limited-stage-diffuse-large-b-cell-lymphoma). A recent clinical trial^35^ (UpToDate 11, https://www.uptodate.com/contents/initial-treatment-of-limited-stage-diffuse-large-b-cell-lymphoma) directly compared the outcomes of R-CHOP (4–6 cycles) with or without subsequent radiation therapy (RT) in limited-stage (Stage I or II) DLBCL patients. The group that received RT had a 5-year OS rate of 96%, while the group that did not receive RT had a 5-year OS rate of 92% (i.e., no statistically significant difference between groups). The median time to relapse was also the same for both groups, as well as cardiac and hematologic toxicity profiles, but three patients in the RT group exhibited symptoms of radiation-induced toxicity. Therefore, the authors of the study recommend withholding RT for limited-stage DLBCL patients who show a complete response (CR) on PET scan after 4–6 cycles of R-CHOP^35^ (UpToDate 11, https://www.uptodate.com/contents/initial-treatment-of-limited-stage-diffuse-large-b-cell-lymphoma). Overall, most patients diagnosed with limited-stage DLBCL have favorable outcomes when treated with R-CHOP, with or without IFRT.

However, the same cannot always be said for patients diagnosed with advanced-stage DLBCL (50–70%, depending on the reference)^2,3^ (UpToDate 1, https://www.uptodate.com/contents/epidemiology-clinical-manifestations-pathologic-features-and-diagnosis-of-diffuse-large-b-cell-lymphoma). There are likely multiple reasons for this fact. First, advanced-stage DLBCL is disseminated throughout the body, affecting multiple lymph node regions and/or organs. Second, advanced-stage DLBCL contains greater genetic and epigenetic (i.e., changes in gene expression that occur without altering the actual DNA sequence) heterogeneity than does limited-stage DLBCL. Third, advanced-stage disease tends to have a higher proportion of patients with ABC-type DLBCL, which is more aggressive and associated with a worse prognosis than the GCB-type^36^ (UpToDate 4, https://www.uptodate.com/contents/prognosis-of-diffuse-large-b-cell-lymphoma). This makes sense, given that the GCB subtype of DLBCL has instead been observed to be more enriched in limited-stage disease^35,36^. And, fourth, the co-expression of *MYC* and *BCL2* (two proto-oncogenes strongly associated with aggressive lymphomas) is more likely in advanced-stage DLBCL and is independently associated with a worse prognosis^36^ (UpToDate 4, https://www.uptodate.com/contents/prognosis-of-diffuse-large-b-cell-lymphoma). A study published in 2015 (ref. ^36^) (UpToDate 4, https://www.uptodate.com/contents/prognosis-of-diffuse-large-b-cell-lymphoma) analyzed biopsies from 344 de novo DLBCL patients (49% limited-stage and 51% advanced-stage) treated with R-CHOP in order to determine molecular subtype and to assess subtype-specific clinical outcomes. Limited-stage patients had a 5-year OS rate of 86% for the GCB subtype and 69% for the ABC subtype, whereas advanced-stage patients’ 5-year OS rates were 74% for the GCB subtype and 51% for the ABC subtype. Outcomes were also determined based on the co-expression of *MYC* and *BCL2*, although these were not stratified by stage. Patients who were not *MYC*+/*BCL2*+ had a 5-year OS rate of 76% (81% for GCB and 62% for ABC), while those who were *MYC*+/*BCL2*+ had a 5-year OS rate of 54% (64% for GCB and 51% for ABC). Taken together, these findings emphasize the need for molecular subtyping of advanced-stage DLBCL in order to predict its clinical outcome and choose the most appropriate treatment. Since the GCB subtype of advanced-stage DLBCL has a *relatively* good prognosis (5-year OS of 74%)^36^ (UpToDate 4, https://www.uptodate.com/contents/prognosis-of-diffuse-large-b-cell-lymphoma), R-CHOP (6 cycles; 21 days between each cycle) is still its standard therapy (UpToDate 5, https://www.uptodate.com/contents/initial-treatment-of-advanced-stage-diffuse-large-b-cell-lymphoma). However, due to the poor outcomes of ABC-type advanced-stage DLBCL and DLBCLs that co-express *MYC* and *BCL2* (without chromosomal translocations) in response to R-CHOP, it is often recommended that these patients enroll in a clinical trial (UpToDate 5, https://www.uptodate.com/contents/initial-treatment-of-advanced-stage-diffuse-large-b-cell-lymphoma).

Overall, while R-CHOP can achieve relatively effective 5-year OS rates in certain subsets of DLBCL patients (e.g., limited-stage and GCB-type), ~30% of all patients either do not respond or relapse within 5 years of treatment^24,25^, and 30–50% of all patients are not cured^26,37^. Furthermore, while some components of this regimen do exhibit slight specificity (Rituximab targets the B-cell marker CD20; Prednisone targets inflammatory pathways and immune cells), even these still affect normal cells, and the regimen as a whole targets rapidly dividing cells indiscriminately. Consequently, recipients of R-CHOP often experience many of the side effects for which chemotherapy is notorious (e.g., hair loss, vomiting, and immune suppression). R-CHOP’s lack of specificity is even more problematic in light of DLBCL’s extensive genetic and epigenetic heterogeneity. In general, as the heterogeneity of a cancer increases, the likelihood that a given treatment will effectively treat all of its subclones decreases. These conditions can result in a poor initial response to therapy and/or the selection of chemoresistant subclones that lead to relapse.

## Natural history and molecular pathogenesis of DLBCL

### Normal B-cell function

To address the shortcomings of standard DLBCL therapy, researchers in the past couple of decades have placed greater emphasis on understanding the natural history and molecular pathogenesis of the disease. Because cancers are derived from normal cells, it would be difficult to fully comprehend the etiology and behavior of DLBCL without first examining normal B-cells and the principles that underlie their function. As the cornerstone of humoral immunity, naive B-cells’ main objectives are to (1) recognize antigens (from pathogens), (2) produce B-cell receptors (BCRs) with high affinity for antigens, and (3) differentiate into either memory B-cells (for faster response to future infections) or plasma cells, which actively secrete high-affinity antibodies. Humoral immunity is essential for fighting infections and is also the biological process that makes vaccination possible. A recent study of circulating B-cells in a cohort of ten human subjects found that the human antibody repertoire may contain as many as 10^16^–10^18^ unique heavy/light-chain combinations^38^. Theoretically, this staggering diversity should enable the body to respond to any foreign antigen that it may encounter. However, B-cells cannot accomplish all of these objectives alone or in their initial state. They must cooperate with dendritic cells and T-cells, progress through a coordinated sequence of modifying events, and survive an intense selection environment, all of which occur during a process known as the GC reaction.

### The GC reaction

**Fig. 2:**
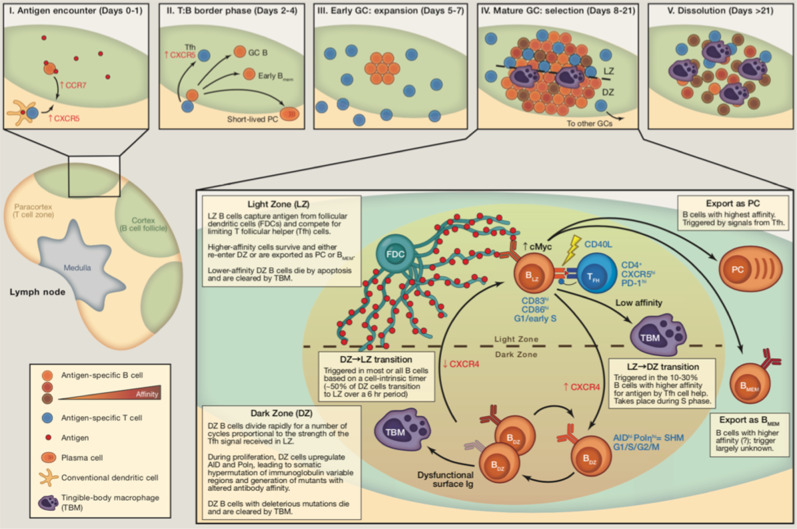
The germinal center (GC) reaction. The GC reaction is the foundation of humoral immunity. Its end products are memory B-cells and plasma cells that encode high-affinity antibodies. However, it also is the source of many types of B-cell lymphoma, including DLBCL. The time-lapse panels at the top of this figure depict the sequential steps of the GC reaction, which take place within lymph node follicles. The large panel at the bottom zooms in to show the mechanisms behind, and outcomes of, the selection of GC B-cells by T_FH_ cells in the light zone of the germinal center (Figure Source: Victora^41^. See this reference for a detailed review).

The GC reaction (Fig. 2) starts when a mature, naive B-cell encounters an antigen in a secondary lymphoid tissue (e.g., lymph node). Although the BCR of this cell does recognize the antigen, its baseline affinity for the antigen is low. The B-cell migrates to the edge of a lymphoid follicle, where it presents a peptide from the antigen to CD4+ T-cells via major histocompatibility complex II (MHC II). This step is selective, as B-cells with higher baseline affinity for antigen than their competitors are preferentially bound by T-cells. After receiving T-cell stimulation, the B-cell starts proliferating and, then, relocates to the center of the follicle, where it seeds the formation of the GC. Once it has matured, the GC contains two distinct compartments: the dark zone (DZ) and the light zone (LZ). The DZ consists of highly proliferative B-cells called centroblasts that divide once every 6–12 h^39^ and undergo random somatic hypermutation (SHM) of the genes encoding the variable regions of immunoglobulins (IgVs). After dividing 1–6 times^40^, centroblasts can transition to the LZ, where the affinity of their newly modified BCR for antigen is tested. The LZ consists of non-replicative B-cells (centrocytes), follicular dendritic cells (FDCs), and T follicular helper (T_FH_) cells. A centrocyte first receives some of the antigen from an FDC, whose tendril-like appendages serve as an antigen reservoir. Once the centrocyte processes the antigen, it presents MHC II loaded with peptide to T_FH_ cells. T_FH_ cells are limited in number, which forces centrocytes to compete with one another. Centrocytes have three potential paths, and each cell’s outcome is entirely dependent upon the affinity of its BCR for antigen(s). The “default setting” of a centrocyte is to undergo apoptosis in the absence of sufficient T-cell signal (i.e., low affinity). Thus, only centrocytes whose BCRs have high affinity for antigen are positively selected by T_FH_ cells. Cells that are positively selected then undergo class switch recombination (CSR) to switch from the default IgM isotype to IgG, IgA, or IgE (depending on context), followed by differentiation into memory B-cells or plasma cells. However, 10–30% of centroctyes^41^ have BCRs with intermediate affinity for antigen, which is enough to interact with T_FH_ cells and avoid apoptosis but not enough for true positive selection. These cells are instead sent back to the DZ for subsequent cell divisions and SHM, and they will later be given another opportunity for positive selection in the LZ. (The above information was adapted from refs. ^39–42^. Refer to these for a more detailed discussion of the topic.)

The GC reaction owes much of its “design” to the principles of biological evolution, as it marries genetic combinatorics (baseline antibody diversity + SHM) with a natural (and clonal) selection environment (positive selection by T_FH_ cells) to find the cells that respond best to a selective pressure (possess BCRs with the highest affinity for an antigen). This strategy also resembles combinatorial optimization, a process used in mathematics and computer science to find the optimal solution to a specific problem from a finite number of potential solutions. Though the number of potential unique antibodies is technically finite, it is so large that it likely would not be feasible to systematically test all of them. As a compromise, different antibodies are modified and tested at random until one (or more) is found that binds a specific antigen with sufficiently high affinity. This mirrors the logic of evolution, by which “survival of the fittest” does not necessarily guarantee the greatest of all possible fits, but rather one that sufficiently overcomes a selective pressure and outperforms its competitors. If applied in the appropriate context and with strict guidelines, these principles can be quite effective for performing a normal biological function like the GC reaction.

However, as those in the field of cancer biology have known for decades, cancer is also driven by evolutionary principles and follows a pattern of clonal selection and evolution^43^. Viewed from this perspective, it is not difficult to imagine how a system that intentionally recreates a Darwinian microenvironment could (1) cause or promote oncogenic events, even under “normal” conditions or (2) become pathologically dysregulated and repurposed as a sort of “operating system” for cancer cells. Furthermore, the physiological mechanisms that the GC reaction requires to function are inherently risky. The transformation from GC B-cells to DLBCL cells often involves the removal of negative feedback and temporal restrictions on normal, essential pathways, without any functional alteration of the protein(s) involved. In other words, simply upregulating the core mechanisms of the GC reaction and/or increasing their duration can sometimes be enough to initiate oncogenesis (as shown in experiments; discussed later). Along these lines, multiple researchers^40,44^ have highlighted the fact that B-cells undergoing the GC reaction naturally exhibit multiple characteristics that resemble the hallmarks of cancer^45^.

### Activation-induced deaminase and the GC reaction

The GC reaction is complex and multifaceted, but there are two components in particular that can explain a great deal about its physiology, the risks inherent to its strategy, and its sometimes-oncogenic side effects. The first component is activation-induced deaminase (AID), an enzyme that deaminates cytosine residues at specific sites throughout the genome. AID is required for both SHM and CSR^46^, which are required for making high-affinity and class-switched antibodies, respectively. This deamination converts cytosines to uracils, which result in U:G mismatches that trigger DNA repair via the mismatch repair (MMR) or base excision repair (BER) pathways. For some genes, especially those that encode IgVs, DNA repair involves an error-prone DNA polymerase (Pol η). This increases the rate of mutations, insertions, and deletions that fuel SHM and the DNA double-stranded breaks (referred to as DNA-DSBs) that are required for CSR (although DNA-DSBs can occur during SHM as well). Perhaps not surprisingly, a mechanism that intentionally causes DNA damage can have serious side effects, and most B-cell lymphomas can be traced back to cells that come from the GC and/or have gone through the GC reaction^47^. The side effects of the GC reaction primarily include chromosomal translocations and oncogenic mutations. Translocations (e.g., *MYC*, *BCL2*, and *BCL6)* are quite common in B-cell lymphomas and NHLs in general. They usually involve the intact coding region of a proto-oncogene being placed under the control of an immunoglobulin gene regulatory sequence (e.g., enhancer), resulting in constitutive expression. While AID is suspected to increase the likelihood of DNA-DSBs and translocations for multiple genes, it has been most firmly linked to *MYC* translocations. In a set of in vivo experiments, Pasqualucci et al. showed that mice engineered to overexpress *BCL6* (master regulator of GCs; see section titled “BCL6 and the GC reaction”) but which had a knockout of the gene that encodes AID (*AICDA*, activation-induced cytidine deaminase) were incapable of producing *MYC-IGH* translocations when stimulated to undergo CSR. On the contrary, mice overexpressing *BCL6* and normally expressing *AICDA* showed an increase in the number of *MYC-IGH* translocations of over tenfold when compared with wild-type mice. Furthermore, only ~14% (1/7) of tumors in *BCL6*-overexpressing/*AICDA*-knockout mice (tumor incidence = 7/29, or ~24%) possessed features of DLBCL, compared to ~69% (11/16) of tumors in *BCL6*-overexpressing/*AICDA*-normal mice (tumor incidence = 16/27, or ~59%)^48^.

As was mentioned previously, SHM of genes encoding IgVs is required for the generation of high-affinity antibodies. During SHM, the mutation rate at IgV loci is ~10^6^ times higher than the spontaneous mutation rate observed in somatic cells^49^. The B-cells that survive SHM and selection during the GC reaction benefit from affinity maturation. Each IgV locus obtains approximately nine mutations, and the affinity of their antibodies increase by about 100-fold^50^. It has been known for quite some time that the AID-mediated process of SHM does not always stay within the confines of IgV loci. This pathological “mistargeting” of SHM is called aberrant SHM (aSHM). aSHM does not occur in normal GC B-cells and is unique to GC-derived B-cell lymphomas, especially DLBCL, of which over 50% of cases show evidence of aSHM in multiple proto-oncogenes^51^. It is still not entirely clear how aSHM occurs. An interesting study by Liu et al. found that AID actually targets a variety of genes located throughout the genome in normal GC B-cells, many of which are not IgV loci. While a few of these genes (e.g., IgVs) are hypermutated due to DNA “repair” with an error-prone DNA polymerase, many other genes receive high-fidelity repair and are left without a trace of AID activity. This led Liu et al. to propose that aSHM may be due to a breakdown in the high-fidelity repair of genes that would otherwise be “protected” under normal circumstances^52^. Since then, knowledge of the genes that are targeted by AID and/or aSHM has continued to expand^53,54^.

One particular gene that has been studied extensively with regard to both SHM and aSHM is *BCL6* (see above in this section and below in section titled “BCL6 and the GC reaction”)^55–58^. For reasons that are not readily apparent, *BCL6* is the most common non-IgV target of SHM^52,53^, with 59–73% of DLBCL cases^55,57^ and even ~30% of normal GC B-cells^57^ showing evidence of SHM in the 5′ noncoding region. Liu et al. showed that, unlike most of the other non-IgV targets of SHM, *BCL6* seems to receive the same error-prone DNA repair as IgV loci. The mutation rate of *BCL6* in wild-type B-cells is almost as high its mutation rate in cells with knockouts of key genes involved in MMR and BER^52^. Mutations in the 5′ noncoding region can cause the deregulated expression of *BCL6*; for instance, in ~13% of DLBCL cases, such mutations interfere with the ability of BCL6 to negatively regulate its own expression^59^. Another ~40% of DLBCL cases involve chromosomal translocations that lead to upregulated *BCL6* expression, with breakpoints typically located in the same 5′ noncoding region^60,61^ (UpToDate 12, https://www.uptodate.com/contents/pathobiology-of-diffuse-large-b-cell-lymphoma-and-primary-mediastinal-large-b-cell-lymphoma). Lastly, the expression and/or activity of *BCL6* can also be indirectly upregulated as a result of mutations in other genes such as *MEF2B* (myocyte enhancer factor 2B) and *FBXO11* (F-box only protein 11). Regardless of the specific mechanism by which it occurs, the deregulation of *BCL6* expression is very common in DLBCL.

### BCL6 and the GC reaction

**Fig. 3:**
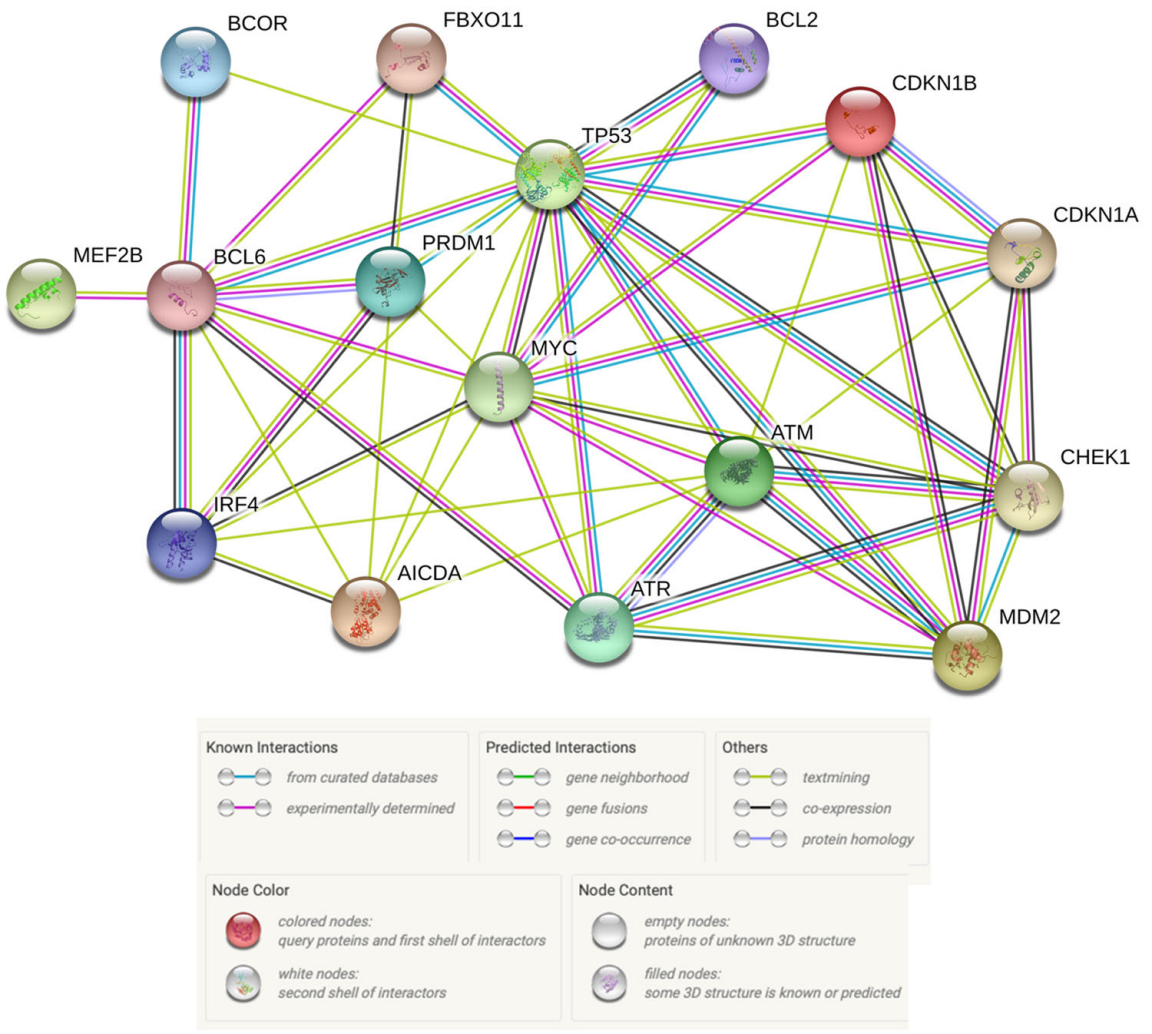
Functional interactions between proteins relevant to GC B-cell and DLBCL physiology. BCOR BCL6 corepressor; FBXO11 F-box only protein 11; BCL2 B-cell CLL/lymphoma 2; TP53 tumor protein 53; CDKN1A cyclin-dependent kinase inhibitor 1A; CDKN1B cyclin-dependent kinase inhibitor 1B; MEF2B myocyte enhancer factor 2B; BCL6 B-cell CLL/lymphoma 6; PRDM1 PR/SET domain 1; MYC myelocytomatosis; ATM Ataxia telangiectasia mutated; ATR Ataxia telangiectasia and Rad3-related protein; CHEK1 checkpoint kinase 1; MDM2 murine double minute 2; IRF4 interferon regulatory factor 4; AICDA activation-induced cytidine deaminase (Figure prepared using: STRING Protein-Protein Interactions Network (https://string-db.org)).

Similarly to AID, the actions of BCL6 are strongly associated with both the GC reaction and the oncogenic transformation of GC B-cells. BCL6 is highly expressed in GC B-cells and is often referred to as the master regulator of the GC reaction. Results from multiple studies have shown that the expression of BCL6 is required for GC formation and antibody affinity maturation^62,63^. BCL6 is a transcriptional repressor whose function is to reduce or prevent the expression of genes whose encoded products would otherwise interfere with the GC reaction (Fig. 3). Recall that GC B-cells must be able to tolerate an enormous amount of DNA damage in order for the processes of SHM and CSR to work, despite the fact that cells are ordinarily on high alert for signs of DNA damage. In GC B-cells, this conflict between preserving genomic integrity and creating high-affinity, class-switched antibodies is mediated in large part by BCL6. BCL6 accomplishes this by repressing the transcription of *TP53* (tumor protein 53), *ATM* (ataxia telangiectasia mutated), *ATR* (ataxia telangiectasia and Rad3 related), *CHEK1* (checkpoint kinase 1), and *CDKN1A* (cyclin-dependent kinase inhibitor 1A, or p21), all of which are critical for signaling and triggering a response to DNA damage, including stopping the cell cycle, initiating high-fidelity DNA repair, and/or inducing apoptosis. BCL6 also represses genes involved in other essential aspects of the GC reaction. These include *PRDM1* (PR/SET domain 1), which is required for the terminal differentiation of GC B-cells into memory B-cells or plasma cells, and the genes encoding microRNAs (miRs) miR-155 and miR-361, which negatively regulate the expression of *AICDA*. Thus, BCL6 and AID can positively regulate each other. (For a thorough review of BCL6’s functions, mechanisms, and targets, see Hatzi and Melnick’s review^44^).

It is not hard to imagine how a system that (1) increases cells’ tolerance of DNA damage, (2) permits rapid progression through the cell cycle, and (3) prevents terminal differentiation, could be compromised and repurposed as a powerful survival mechanism for cancer cells. Using a mouse model engineered to constitutively express *BCL6*, Cattoretti et al. found evidence in support of BCL6’s role in the pathogenesis of DLBCL^64^. Compared to wild-type mice, *BCL6*-overexpressing mice had a significantly greater number of GCs in splenic tissue after immunization and produced ~30% fewer plasmacytoid cells (mostly post-CSR plasma cells), indicating altered post-GC differentiation. By 6 months of age, 42% of the *BCL6*-overexpressing mice developed a benign lymphoproliferative disease, compared to 11% in wild-type mice. At 13 months of age, the *BCL6*-overexpressing mice started to exhibit increased mortality. Between 15 and 20 months of age, 36–62% of *BCL6*-overexpressing mice had developed B-cell lymphoma (compared to 2–8% in wild-type mice), and 75% of these cases resembled DLBCL. Overall, by 20 months of age, 76–89% of *BCL6*-overexpressing mice had either lymphoproliferative disease or lymphoma (compared to 8–14% in wild-type mice), as well as a significant decrease in survival^64^.

It is striking that this same protein can be both required for the GC reaction and a driver of GC-derived lymphoma, simply by deregulating its expression without any functional alteration. It seems that this system has evolved with an “awareness” of the oncogenic potential of BCL6, as it is normally restrained by multiple regulatory mechanisms. BCL6 can inhibit its own expression via negative feedback (see section titled “AID and the GC reaction”), and it is also downregulated and targeted for degradation by multiple signaling pathways in response to high-affinity BCR-antigen binding and positive selection by T_FH_ cells^42,65,66^. The expression of *BCL6* must be shut off and is not normally expressed in post-GC cells, because one of its main targets, *PRDM1*, is required for GC exit and differentiation. Interestingly, BCL6 also represses the transcription of a number of proto-oncogenes, including *MYC* and *BCL2* (ref. ^67^). MYC is critical for cyclic reentry of LZ centrocytes back into the DZ for further replication and SHM. BCL2 is an anti-apoptotic factor that further increases the threshold for programmed cell death. In their review of BCL6, Hatzi and Melnick propose that the repression of *MYC* and *BCL2* by BCL6 may be an attempt to compensate for its repression of tumor suppressor genes^44^. It may also partly explain why *MYC* and *BCL2* translocations are not uncommon in DLBCL, as they allow escape from BCL6 repression^44^.

### The genetic heterogeneity of DLBCL

As one might expect of a cancer derived from cells and an environment centered around combinatorial diversity, heterogeneity is a defining characteristic of DLBCL. This can be observed all the way from its clinical outcome, to its cellular morphology and phenotype, and down to its molecular profile, where DLBCL demonstrates a staggering amount of genetic and epigenetic heterogeneity. Starting at the genetic level, numerous studies over the past decade have analyzed hundreds of DLBCL patients’ tumor genomes in an effort to better understand the molecular pathogenesis of the disease^15,16,68–73^. Mutations have been found in more than 700 different genes^39^, with each case of DLBCL having an average of 50–100 genetic lesions in the coding genome^69,71,72,74^. Approximately 150 of these genes are mutated in >5% of patients and considered genetic drivers of DLBCL, with an average of ~8 driver mutations per case^73^. A genomic analysis of more than 92,000 cases of over 100 types of cancer found that DLBCL had the fourth-highest tumor mutational burden (TMB), with a median of 10 mutations per megabase (Mb) of DNA and ~18% cases with >20 mutations/Mb^75^. Of the ten cancer types with the highest TMB in this study, DLBCL (#4) and FL (#8; can transform into DLBCL) are the only ones which are not epithelial (i.e., carcinomas) or melanocytic (i.e., melanoma) in nature. Furthermore, all other cancers in the “top 10” besides DLBCL and FL are derived from the lung, skin, or melanocytes, all of which are highly associated with exogenous sources of DNA damage (e.g., smoking and UV light)^75^. This further corroborates the notion that the occurrence of DLBCL is largely due to endogenous (but still powerful) sources of DNA damage^76^.

### Endogenous sources of mutation and their signatures

Chapuy et al. recently analyzed the mutational signatures in the genomes of 304 DLBCL patients’ tumors in an attempt to identify the source(s) of mutations in recurrently affected genes^16^. Approximately 80% of all mutations were linked to the spontaneous deamination of cytosines at CpGs and a switch from cytosine to thymine (C > T). This type of mutation is associated with aging and, accordingly, the tumors of older patients in this cohort had more mutations with this signature than did younger patients’ tumors. This finding also aligns with the fact that DLBCL patients are usually diagnosed at an older age (median 66 years old) (SEER3, https://seer.cancer.gov/statfacts/html/dlbcl.html). Two other mutational signatures were prominent in this study as well. One signature, termed “canonical AID (cAID),” was linked to an increase in C > T/G mutations at AID hotspots and was associated with both SHM and aSHM^77^. The other, termed “AID2,” had similarities to a non-canonical AID signature that is linked to an increase in A > T/C/G mutations and is associated with error-prone DNA repair subsequent to cytosine deamination by AID^77^. The contribution of each mutational process varied depending on the particular gene, with some genes (e.g., *BCL2*) being mutated mostly by the cAID and AID2 processes, and other genes (e.g., *NOTCH2*) being mutated almost exclusively by “aging”. It is interesting to note that these two AID mutational signatures are similar to the ones initially described by Liu et al.; one involves high-fidelity DNA repair, and the other involves error-prone DNA repair^52^. It should also be noted that mutations caused by spontaneous deamination (C > T) and cAID (C > T/G) can remove methylated cytosines, thereby reducing DNA methylation and altering cells’ epigenetic profiles. Multiple studies have even implicated AID-induced DNA demethylation as a means of epigenetic reprogramming that promotes pluripotency^78–81^. The relationship between AID and DNA methylation will be revisited in the section titled “Connections between epigenetic dysregulation and relapse in DLBCL”. Presently, however, a broader discussion of the epigenetic landscape in DLBCL is warranted.

### Frequent mutations in genes related to chromatin and epigenetics

**Fig. 4:**
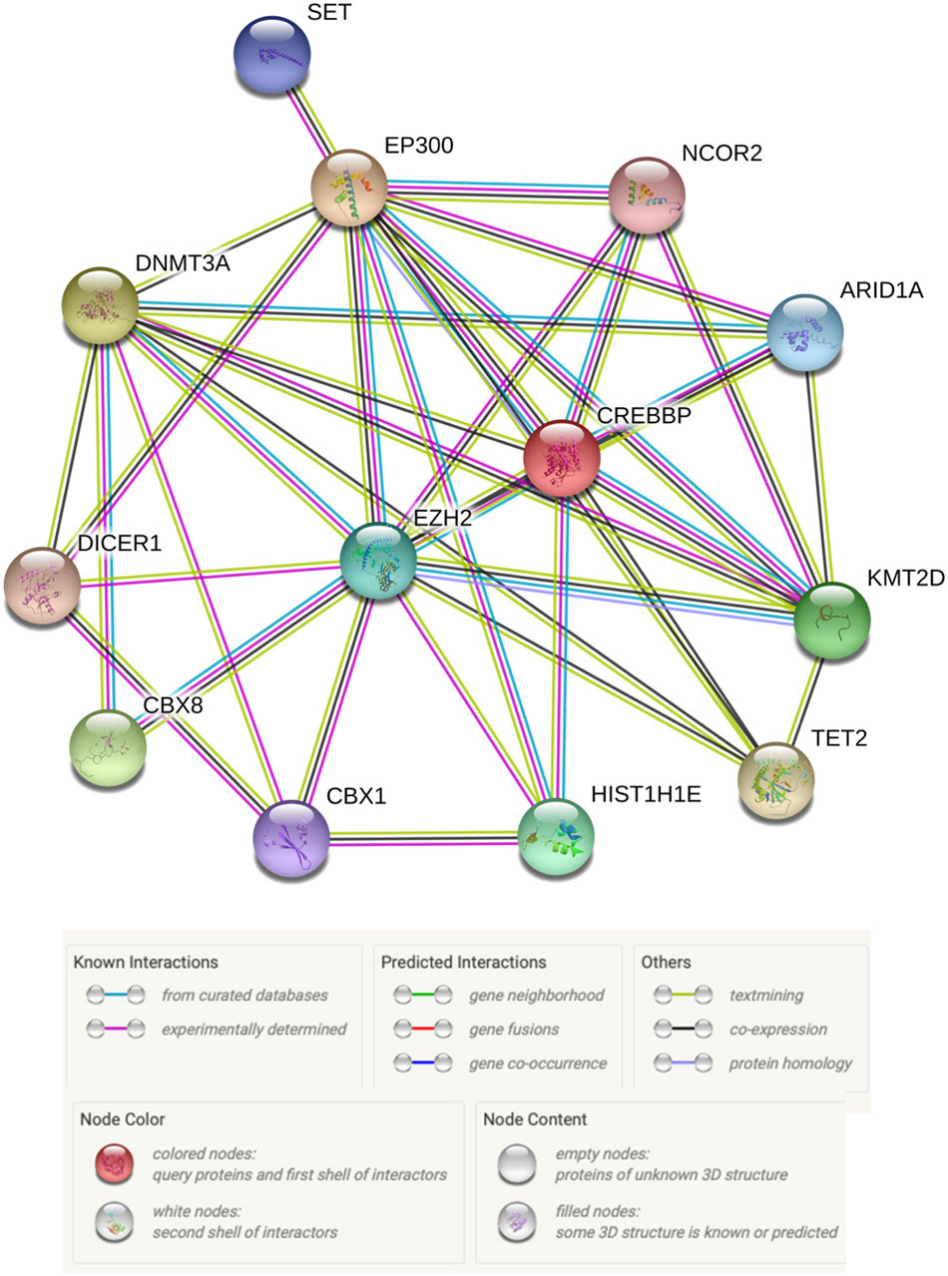
Functional interactions between proteins relevant to GC B-cell and DLBCL epigenetics. SET SET nuclear proto-oncogene; EP300 E1A binding protein 300; DNMT3A DNA methyltransferase 3A; NCOR2 nuclear receptor corepressor 2; ARID1A AT-rich interaction domain 1A; CREBBP CREB-binding protein; EZH2 Enhancer of zeste 2; DICER1 Dicer 1, ribonuclease III; CBX8 Chromobox 8; CBX1 Chromobox 1; HIST1H1E histone cluster 1 H1 family member E; TET2 Tet methylcytosine deoxygenase 2; KMT2D lysine methyltransferase 2D (Figure prepared using: STRING Protein-Protein Interactions Network (https://string-db.org)).

One of the most consistent trends that has emerged from genomic analyses of DLBCL is the recurrence of mutations in genes whose products are specifically related to chromatin and epigenetics (Fig. 4). Of the 150 genetic drivers of DLBCL described by Reddy et al., 21 (i.e., 14%) of them fit this description (#1 *MLL2*; #4 *HIST1H1E*; #6 *CREBBP*; #10 *ARID1A*; #15 *ARID1B*; #16 *SETD1B*; #18 *SMARCA4*; #33 *SETD2*; #34 *TET2*; #37 *ARID5B*; #38 *EZH2*; #43 *EP300*; #44 *MLL3*; #54 *INO80*; #55 *CHD8*; #58 *DNMT3A*; #71 *NCOR1*; #75 *CHD1*; #88 *SETD5*; #115 *DICER1*; #128 *HIST1H2BC*)^73^. This subset of genes is skewed toward the top of the list, with 18/21 (86%) located in the “top 75,” 7/21 (33%) in the “top 20,” and 4/21 (19%) in the “top 10,” including the #1 most commonly altered gene in DLBCL: *MLL2* (alternative name for *KMT2D* (lysine methyltransferase 2D)). The functions of these genes include regulation of DNA methylation (e.g., *DNMT3A*) and demethylation (e.g., *TET2*), miRNA processing (e.g., *DICER1*), chromatin remodeling (e.g., *ARID1A*), linker histone-mediated chromatin compaction (e.g., *HIST1H1E*), and post-translational modification (PTM) of histones (e.g., *KMT2D*). This last functional category, consisting of histone-modifying enzymes, are some of the most common mutations in DLBCL^69,70,73^ and are integral to both the physiology of the GC reaction and the molecular pathogenesis of DLBCL.

As was just stated, *KMT2D* (sometimes called *MLL2* or *MLL4*) is the most commonly mutated gene in DLBCL, with ~25% of cases showing genetic alteration^73^. KMT2D is a histone methyltransferase that is primarily responsible for the monomethylation of lysine 4 of histone H3 (H3K4me1), an epigenetic mark that is associated with active gene enhancers. *CREBBP* (CREB-binding protein; ~11%) and *EP300* (E1A binding protein 300; ~6%) are genetically altered in ~17% of DLBCL cases, usually in a mutually exclusive fashion due to their high structural and functional homology (although *CREBBP* mutations are more frequent)^73^. These two genes encode the eponymous lysine acetyl transferases (KATs; previously called histone acetyltransferases (HATs)) that acetylate lysines 18 and 27 of histone H3 (H3K18Ac and H3K27Ac), the latter of which is required for gene enhancer activation. CREBBP and EP300 also acetylate a variety of non-histone targets, including BCL6 and p53; the significance of this will be revisited later (see section titled “The epigenetic switch at enhancers”).

*EZH2* (Enhancer of Zeste 2), the enzymatic subunit of PRC2 (polycomb repressor complex 2), is a histone methyltransferase that is responsible for the mono-, di-, and trimethylation of lysine 27 of histone H3 (H3K27me1/2/3), all of which contribute to regulating promoters’ availability to transcriptional machinery. About 6% of all DLBCL cases feature mutations in *EZH2* (ref. ^73^). However, unlike mutations in *KMT2D*, *CREBBP*, and *EP300* (refs. ^68,70^), mutations in *EZH2* only occur in the GCB subtype, not the ABC subtype. Within the GCB subtype specifically, as many as ~22% cases possess an *EZH2* mutation^82^. Mutations in *EZH2* are always heterozygous, and they almost always target tyrosine residue 641 (Y641) within the enzyme’s catalytic site^82^. This alters the enzymatic activity of *EZH2* and causes it to favor the trimethylation of H3K27 over mono- or dimethylation. The remaining wild-type *EZH2* allele is responsible for providing most of the H3K27 mono- and dimethylation that cells still require and probably explains why *EZH2* mutations are exclusively heterozygous in DLBCL^83^.

Referring back to the analysis of mutational signatures in DLBCL by Chapuy et al., it is interesting to note that these mutations in histone-modifying genes are enriched in the “aging” signature, albeit to varying degrees. Approximately 90% of *KMT2D* and *EP300* mutations in their dataset were linked to aging, as well as ~70% of *CREBBP* mutations and >50% of *EZH2* mutations^16^. The potential implications of this finding will be discussed later on in this review. (See sections titled “Endogenous sources of mutations and their signatures” and “Connections between epigenetic dysregulation and relapse in DLBCL”.)

## Epigenetics determinants of DLBCL

### Epigenetic switches in GC B-cells

It has been established that B-cells must progress through a specific sequence of steps during the GC reaction. Some of these steps, such as the transition from naive B-cell to centroblast in the DZ, are straightforward and proceed only in one direction. Other steps, however, require decisions to be made and have multiple potential outcomes. For instance, as centroblasts move from the DZ to the LZ, they face the decision of either going through CSR and becoming plasmacytes or returning to the DZ for subsequent rounds of cell division and SHM. Some GC B-cells have to cycle between the DZ and LZ multiple times before they are allowed to proceed to CSR. Furthermore, once centrocytes go through class-switching and exit the GC reaction as plasmacytes, they still must commit to differentiation and decide whether to become memory B-cells or plasma cells. All of these transitions and decisions require specific alterations in cellular function and identity, which are made possible by rapid and highly coordinated changes in the expression of particular subsets of genes. This is especially true for GC-B cells that cycle between the DZ and LZ, as they must be able to switch between the gene expression patterns that distinguish centroblasts from centrocytes at will, and sometimes repeatedly. GC B-cells achieve this level of plasticity using a system of epigenetic “switches” that govern the GC reaction through the addition or removal of specific histone PTMs at the promoter or enhancer sequences of genes that require up- or downregulation (for an in-depth discussion, see Jiang and Melnick’s review^84^).

Critically, the addition and removal of histone PTMs are reversible, which allows cells to “toggle” between “on” and “poised” (i.e., temporarily off, or paused) states of gene expression efficiently and without fully repressing genes that may soon be needed again. The two epigenetic switches, one located at promoters and the other at enhancers, are essential for the normal physiological function of GC B-cells and are also very commonly dysregulated during the pathogenesis of DLBCL. In fact, many of the proteins that are responsible for maintaining and operating the two epigenetic switches are encoded by genes that are some of the most frequently mutated in DLBCL, including *KMT2D*, *CREBBP*, *EP300*, and *EZH2*. Additionally, the all-important BCL6 exerts its repressive effects at the promoters or enhancers of target genes by forming complexes with histone-modifying enzymes that alter the epigenetic landscape. Thus, the molecular pathogenesis of DLBCL can be better understood by examining these epigenetic switches and how they are differentially regulated in normal versus pathological conditions.

### The epigenetic switch at promoters

**Fig. 5:**
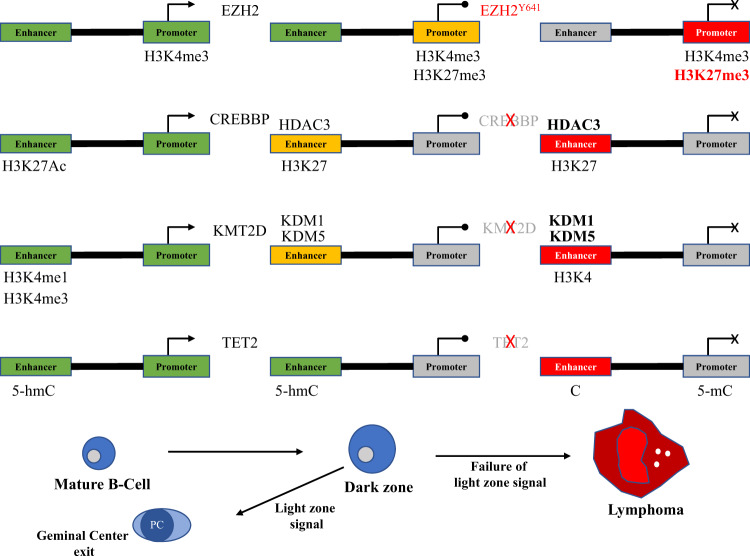
Epigenetic switches at promoters and enhancers in GC B-cells. Starting, maintaining, and exiting from the GC reaction requires rapid and coordinated changes in the expression of specific subsets of genes in response to cell signals. This is achieved by using epigenetic switches at the promoters and enhancers of these genes. (A) H3K4me3 at a promoter signifies that it is “on” (green). The addition of H3K27me3 by EZH2 switches it to a “poised” (yellow) state of transient repression. The Y641 *EZH2* mutation increases H3K27me3 deposition and turns the promoter “off” permanently (red). (B) H3K27Ac at an enhancer means that it is active. Removal of H3K27Ac by HDAC3 (complexed with BCL6-SMRT) leaves only H3K4me1 marks behind and poises the enhancer. Inactivation of *CREBBP* switches enhancers off by preventing their reactivation via H3K27 (and BCL6) acetylation, leaving HDAC3 unopposed. (C) H3K4me1 is also present at active enhancers. The lysine demethylases KDM1 and KDM5 are thought to remove H3K4 methylation and poise enhancers. *KMT2D* inactivation silences enhancers by preventing the addition of H3K4me1. (D) TET2 demethylates cytosines at enhancers, first by converting 5-methylcytosine (5mC; repressive) to 5-hydroxymethylcytosine (5hmC; active). *TET2* inactivation switches enhancers off by preventing demethylation and, instead, causing hypermethylation. Genes whose promoter and/or enhancer cannot be reactivated makes them unresponsive to important cell signals. This locks cells into the GC reaction, which can lead to lymphomagenesis (Figure based on work by Mlynarczyk et al.^40^).

During the GC reaction, BCL6 represses the expression of over 1000 genes in order to avoid triggering cell-cycle inhibition, apoptosis, or differentiation before the process of affinity maturation is complete^40,44^. BCL6 can repress the expression of a gene by binding either its promoter or enhancer, but the sets of genes that are affected by binding at either location are largely non-overlapping. The precise mechanism by which BCL6 represses gene expression also differs depending on whether it is acting at a promoter or an enhancer^85^. BCL6 does not act alone. Rather, it must collaborate (either directly or indirectly) with other proteins in order to exert its repressive effects, many of which are histone-modifying enzymes. With regard to the epigenetic code, the promoter of a gene is “on” when the nucleosomes packaging the promoter sequence are decorated with H3K4me3 but not H3K27me3. Conversely, when these nucleosomes are decorated with H3K27me3 but not H3K4me3, the promoter is considered “off.” When these nucleosomes possess both H3K4me3 and H3K27me3 (i.e., bivalent chromatin), the promoter is “poised” (Fig. 5).

Referenced earlier (see section titled “Frequent mutations in genes related to chromatin and epigenetics”), the histone methyltransferase EZH2 is required for GC formation and affinity maturation^86^. The main role of EZH2 in the GC reaction is the de novo deposition of H3K27me3 at the promoters of specific target genes that are already marked with H3K4me3 (i.e., active), thereby forming bivalent promoters. Just like BCL6, some of the key genes that EZH2 targets include *PRDM1* and *IRF4*, which are required for the differentiation of GC B-cells into memory B-cells and plasma cells, as well as *CDKN1A* and *CDKN1B*, which are cell-cycle inhibitors^86,87^. There is significant overlap between the gene sets that EZH2 and BCL6 target^88,89^. Based on their experiments, Béguelin et al. recently proposed a model whereby BCL6 and EZH2 collaborate in the repression of common target genes by acting jointly at promoters^89^. Initially, BCL6 binds its target sequence within the promoter, and EZH2 (as part of PRC2) independently increases the level of H3K27me3 at the promoter. The CBX8 (chromobox 8) subunit of PRC1 (polycomb repressor complex 1) then binds to H3K27me3, as PRC1 is typically the protein complex that is responsible for H3K27me3-mediated transcriptional repression. However, in GC B-cells, many of the canonical components of PRC1 are downregulated while other, non-canonical components such as BCOR (BCL6 corepressor) are upregulated. When CBX8 (as part of PRC1) binds to H3K27me3, it brings the PRC1–BCOR complex along with it. This promotes the interaction of BCOR and BCL6 to form a BCL6–BCOR complex. While BCL6 and EZH2 act on the promoter independently and do not make physical contact, their simultaneous action allows for a “combinatorial tethering” that is required for the stable binding of BCOR to BCL6. Stable formation of the BCL6–BCOR complex at poised promoters is what allows the expression of these genes to be temporarily repressed^89^.

Previous experiments by Hatzi et al. showed that BCL6 is also capable of forming a ternary complex with both BCOR and SMRT (silencing mediator of retinoid and thyroid hormone receptors; also called NCOR2, or nuclear corepressor 2) at the promoters of certain genes in DLBCL cells^85^. This BCL6–SMRT–BCOR complex was shown to more strongly repress the expression of target genes than the BCL6–BCOR complex, but it was bound to the promoters of far fewer genes (*n* = 341) than the BCL6-BCOR complex (*n* = 1783). Furthermore, a principal component analysis (PCA) determined that BCL6 actively repressed promoters only when it was bound to BCOR and SMRT (i.e., ternary complex) and when accompanied by a particular chromatin signature (decreased H3K4me3, H3K36me3, H3K79me2, and H3K9Ac; increased H3K27me3 and DNA methylation) that is associated with pausing of RNA polymerase II elongation^85^.

In cases of DLBCL that feature constitutive expression of BCL6 and a mutation in EZH2 that increases its deposition of H3K27me3 (e.g., Y641), the resulting state of repression becomes permanent rather than transient. While it is necessary to temporarily repress the expression of genes whose products would interfere with the GC reaction, prolonging their repression indefinitely greatly increases the risk of oncogenesis. Béguelin et al. observed this in their experiments when they bred mice that constitutively express *BCL6* and possess the *EZH2* Y641 mutant allele^89^. The bone marrow of these mice, as well as that of three different types of control mice, were then transferred to four separate groups of lethally irradiated mice. The mice who received bone marrow from the *BCL6*-overexpressing, *EZH2*-mutant donors experienced a dramatic acceleration in their mortality compared to all of the other mice. The *BCL6*-overexpressing, *EZH2*-mutant recipients also displayed clear evidence of either lymphoma (10/12; FL or DLBCL) or pre-neoplastic lymphoid neoplasia (2/12) upon both gross pathological and histopathological inspection, while none of the mice from the other three groups (wild-type controls (0/4), *EZH2*-mutant only (0/4), and *BCL6*-overexpressing only (0/5)) showed any evidence of disease^89^.

### The epigenetic switch at enhancers

Just like promoters, enhancers also have an epigenetic code that influences their level of activity. An enhancer is only considered “on” when nucleosomes are decorated with H3K27Ac, although H3K4me1 is typically found at active enhancers as well^90^. An enhancer that loses H3K27Ac but retains H3K4me1 is considered “poised.” And, an enhancer that has neither H3K27Ac nor H3K4me1 marks is considered “off” (Fig. 5). CREBBP and P300 are responsible for depositing H3K27Ac at enhancers, which activates them. CREBBP/P300 and BCL6-SMRT compete with each other at enhancers and can co-occupy enhancers, and upregulation of BCL6 is associated with a decrease in P300 binding at enhancers in GC B-cells^85^. BCL6 arguably plays a more direct role in the repression of enhancers, and it is bound to more enhancers than promoters in GC B-cells^85^. When BCL6 binds to its target sequence at an enhancer, it must form a complex similar to the one that it does at promoters in order to enact its repression. However, unlike at promoters, BCL6 only binds SMRT and does not bind BCOR, which is structurally unrelated to SMRT and binds through a different peptide sequence^85,91,92^. Critically, the SMRT corepressor is bound to histone deacetylase 3 (HDAC3), which deacetylates H3K27 and switches active enhancers to a poised state with only H3K4me1 marks remaining^85^.

Subsequent experiments by Jiang et al. investigated the effects that inactivating CREBBP mutations have on the “balance of power” between CREBBP/P300 and the BCL6-SMRT-HDAC3 complex at the enhancers of certain genes in GC B-cells^93^. Their results show that CREBBP inactivation prevents the deposition of H3K27Ac at enhancers that are poised (i.e., have had H3K27Ac removed) during the GC reaction, which prevents both enhancer reactivation and proper expression of the genes that they regulate. Jiang et al. also show that, due to its removal of CREBBP-mediated acetylation, the action of HDAC3 at enhancers is required to properly initiate the GC reaction. In the absence of H3K27 acetylation by CREBBP, HDAC3 activity is left unopposed, and DLBCL cells become HDAC3-dependent for survival. When DLBCL cells with knocked-down *CREBBP* expression were treated with a selective HDAC3 inhibitor, H3K27 acetylation at the enhancers of multiple MHC II genes was rescued. The subsequent expression of those MHC II genes was also rescued. It should also be noted that wild-type CREBBP/P300 can acetylate BCL6 as a form of negative regulation by preventing its association with HDACs^94^. Conversely, acetylation of p53 by wild-type CREBBP/P300 is a form of positive regulation, as it prevents the ubiquitination of p53 by murine double minute 2 (MDM2)^95^. The ability of CREBBP/P300 to acetylate both BCL6 and p53 is strongly inhibited by mutations that inactivate their HAT domain^68,94^. Thus, in addition to the loss of regulatory influence at enhancers that accompanies CREBBP/P300 inactivation, GC B-cells also lose their ability to directly regulate p53 and BCL6, both of which are integral to GC physiology and DLBCL pathology.

Importantly, the subset of enhancers that are affected by CREBBP inactivation strongly overlaps with the subset of enhancers that are bound by the BCL6–SMRT–HDAC3 complex. Some of the most notable genes whose enhancers were affected by CREBBP inactivation (and, thus, unopposed HDAC3 repression) in these experiments are those involved in GC exit and termination, plasma cell differentiation, and MHC II antigen processing and presentation^93^. The impact of CREBBP inactivation on these particular pathways is further corroborated by experiments independently conducted by Zhang et al.^96^. Their results show that *Crebbp* deletion in murine GC B-cells results in decreased expression of certain genes that, in human GC B-cells, are also (1) expressed, (2) marked with H3K27Ac, and (3) bound by CREBBP. Many of these genes are related to signaling pathways (e.g., BCR, CD40, NF-kB, chemokines, cytokines, and lymphocyte migration) that are activated in the LZ^96^. As was reviewed earlier (see section titled “The GC reaction”), the LZ is where B-cells whose BCR affinity for antigen is sufficiently high can be directed to go back to the DZ for further modification or to differentiate into memory B-cells or plasma cells. Furthermore, their data from ChIP-seq (chromatin immunoprecipitation followed by DNA sequencing) experiments demonstrate the same dynamic of CREBBP and BCL6 opposition at the promoters and/or enhancers of genes that are strongly related to initiating, maintaining, and/or exiting the GC reaction. These include genes involved in the cell cycle, responding to DNA damage, apoptosis, differentiation, and multiple signaling pathways, such as BCR, NF-kB, Toll-like receptor (TLR), interferon (IFN), and activation by T-cells^96^.

Taken together, all of these findings depict a scenario in which GC B-cells that possess inactivating CREBBP/P300 mutations can become locked into the GC reaction and unresponsive to the signals that would normally terminate the GC reaction and determine their fate. Those genes whose expression is incompatible with successful affinity maturation, namely those required for DNA damage response, apoptosis, immune recognition, GC exit, and differentiation, cannot be switched back on due to an inability to restore proper H3K27 acetylation at enhancers. In this state, GC B-cells are denied the opportunity to differentiate and perform their intended biological function, while continuing to be exposed to highly mutagenic and potentially oncogenic internal conditions.

### Functional effects of *CREBBP* deficiency

Multiple studies, including some of those discussed above, have documented the functional effects of *CREBBP* deficiency and its contribution to lymphomagenesis. For instance, Zhang et al.^96^ compared the ability of murine splenic B-cells with wild-type, heterozygous, or homozygous-deleted *Crebbp* to terminally differentiate ex vivo. After stimulating cells with lipopolysaccharide (LPS) and interleukin 4 (IL4), flow cytometry showed that ~8.5% of wild-type B-cells exhibited a plasmablastic phenotype (pre-plasma cell; high CD138 and low B220), while only ~3% of B-cells with a heterozygous or homozygous deletion of *Crebbp* showed the same plasmablastic flow cytometry signature. Results from qRT-PCR experiments also showed a significant decrease in *Prdm1* (required for terminal differentiation) expression in *Crebbp*-deleted (hetero- and homozygous) vs. wild-type cells. Flow cytometry of splenic B-cells taken from a separate cohort of mice after immunization with sheep red blood cells (SRBCs) also showed a significant decrease in the percentage of high-CD138/low-B220 B-cells in *Crebbp*-deleted (hetero- and homozygous) vs. wild-type mice. Additional flow cytometry experiments demonstrated that *Crebbp*-deleted (hetero- and homozygous) splenic B-cells showed higher levels of proliferation and viability as well when compared to wild-type cells. Lastly, they investigated the direct effect of *Crebbp* deletion on the lymphomagenesis in mice in vivo. Deletion of *Crebbp* on its own was not enough to cause a statistically significant increase in lymphoma incidence, although 3/22 cases in the heterozygous knockout group (vs. 0/20 in wild-type and 0/24 in homozygous knockout groups) did develop lymphoma (two DLBCL and one FL). However, in mice with both a heterozygous *Crebbp* deletion and deregulated *Bcl2* expression (which frequently co-occur in FL and DLBCL), there was a significant increase in lymphoma incidence, with 92% (22/24) of these mice developing some type of FL, compared to 61.5% (16/26) in mice with deregulated *Bcl2* expression but wild-type *Crebbp*^96^.

Likewise, Hashwah et al. investigated the effects of *CREBBP* mutation/deletion on GC B-cell proliferation and lymphomagenesis^97^. Starting with in vitro experiments, CRISPR (clustered regularly interspaced short palindromic repeats) was used to introduce an inactivating mutation to one *CREBBP* allele in a wild-type human DLBCL cell line. While this did result in decreased H3K18 acetylation and changes in gene expression, especially those involved in MHC II antigen processing and presentation, no significant difference in growth rate as a function of *CREBBP* status was observed. However, when human DLBCL cells with wild-type or heterozygous-mutant *CREBBP* were subcutaneously xenografted onto mice, the tumors consisting of *CREBBP*-mutant cells grew faster and had a greater mass than tumors of wild-type *CREBBP* cells. Moreover, orthotopic xenografts established intravenously using the same human DLBCL cells with wild-type or heterozygous-mutant *CREBBP* showed a greater capacity for engraftment in the bone marrow of both immunocompromised mice and mice with a humanized immune system. In separate experiments, groups of mice were engineered to delete one or both alleles of *Crebbp* or *Ep300* in response to AID activity after SRBC immunization^97^. Heterozygous and homozygous *Crebbp*-deleted mice exhibited hyperproliferation of GC B-cells while, interestingly, the opposite occurred for mice with heterozygous and homozygous deletions of *Ep300*. Histopathological analysis of splenic tissue showed that the increase or decrease in the number of GC B-cells was due to an increase or decrease of the size of GCs, respectively. Lastly, they also wanted to assess the extent to which loss of *Crebbp* contributes to lymphomagenesis. Similar to the findings of Zhang et al.^96^, heterozygous *Crebbp* deletion was not sufficient to induce lymphomagenesis by itself. However, mice with both a heterozygous deletion of *Crebbp* and constitutive expression of *Myc* developed lymphoma earlier (~20 days post-immunization) and had worse OS (12.5%; 1/8) than mice with wild-type *Crebbp* and constitutive *Myc* expression (~60 days post-immunization; 50%; 2/4)^97^.

Finally, in addition to the more mechanistic experiments described earlier in this section, Jiang et al. studied the functional effects of *Crebbp* loss^93^. Hematopoietic progenitor cells (HPCs) were isolated from mice with deregulated *Bcl2* expression, transduced with either a control retrovirus or one that expresses anti-*Crebbp* shRNA (short hairpin RNA), and then transplanted to lethally irradiated wild-type mice. The lymphomas of mice that received HPCs with knocked-down expression of *Crebbp* displayed an earlier onset and a more aggressive, invasive phenotype than lymphomas with normal *Crebbp* expression. A similar phenotype was also observed when the same experiments were performed with *Ep300* knockdown^93^. In summary, the results of these functional studies support a role for the loss of *CREBBP* in promoting increased GC B-cell proliferation, GC expansion, decreased terminal differentiation, greater aggression and invasiveness, and an increased capacity for lymphomagenesis when combined with the deregulated expression of known oncogenes. In many instances, the loss of *EP300* has similar effects, although this is not always the case.

### Functional effects of *KMT2D* deficiency

It was alluded to previously (see section titled “The epigenetic switch at enhancers”) that, while the presence or absence of H3K27Ac at nucleosomes along an enhancer is what ultimately distinguishes between its “on” and “off” states, respectively, H3K4me1 is also typically present at active enhancers. Furthermore, in the absence of H3K27Ac, the presence of H3K4me1 at an enhancer signifies that it is in a “poised” state rather than completely “off.” KMT2D is responsible for the mono-, di-, and trimethylation of H3K4, which it accomplishes through its catalytic SET (Su(var)3–9, Enhancer-of-zeste, and Trithorax) domain. Since *KMT2D* is the most commonly mutated gene in DLBCL, multiple investigations have been conducted in order to better understand the molecular and functional effects of KMT2D mutations, as well as their impact on lymphomagenesis.

Zhang et al. compared the in vitro methyltransferase activity of 16 different KMT2D mutants derived from DLBCL^98^. Eleven mutants showed a significant decrease in activity, most of which (9/11) had a mutation close to the SET domain and were among the most severely affected. These results were validated in vivo by measuring genome-wide levels of H3K4me1, H3K4me2, and H3K4me3 in splenic B-cells from mice with wild-type, heterozygous-deleted, or homozygous-deleted *Kmt2d*. Heterozygous mice showed a small increase in all three (H3K4me1/2/3), while those with a complete loss of *Kmt2d* showed a sharp decrease in H3K4me1/2/3. A similar pattern was observed in a panel of human DLBCL cell lines, except that heterozygous cell lines also showed a decrease in H3K4me1/2/3. Mice with homozygous loss of *Kmt2d* also experienced changes in B-cell development after SRBC immunization, including significantly fewer B220+ B-cells in lymphoid tissues, mature B-cells in the bone marrow, and follicular B-cells in the spleen. The formation of GCs was also affected in SRBC-immunized mice with homozygous loss of *Kmt2d*. These mice exhibited significant increases in their GC B-cell population and the number, average size, and total area of GCs. The results in mice with a heterozygous deletion of *Kmt2d* were similar to those of mice with complete *Kmt2d* loss but were generally of lesser magnitude. The relative depletion of cells that precede GC B-cells (e.g., follicular B-cells) and expansion of GC B-cells and GCs that occur in these mice suggest that loss of *Kmt2d* encourages mature B-cells to enter the GC reaction more readily. Separate ex vivo experiments also showed that splenic B220+ B-cells taken from *Kmt2d*-deficient mice also display a greater proliferative rate than wild-type cells. This observation fits with results from gene expression analyses showing that the transcriptional signature in cells that have lost *Kmt2d* is enriched in genes that are involved in cell-cycle regulation and apoptosis^98^. Lastly, the extent to which *Kmt2d* loss directly influences lymphoma incidence and pathogenesis was tested in mice with wild-type, heterozygous-deleted, and homozygous-deleted *Kmt2d*. Similar to the findings of Zhang et al. and Jiang et al. in their studies of *CREBBP*^93,96^, deletion of *Kmt2d* alone was not enough to significantly increase lymphomagenesis (0/23 wild-type, 0/22 heterozygous-deleted, and 0/15 homozygous-deleted mice). However, in mice with both loss of *Kmt2d* and deregulated *Bcl2* expression, lymphoma incidence increased from 44.4% (12/27) in wild-type mice to 62.5% (15/24) in heterozygous mice and 78.6% (22/28) in homozygous mice, with cases ranging from early FL to DLBCL^98^.

In an independent study, Ortega-Molina et al. also investigated the influence of *Kmt2d* deficiency on lymphomagenesis in multiple mouse models^99^. First, HPCs obtained from mice with deregulated *Bcl2* expression were transduced with either a control or anti-*Kmt2d* shRNA-expressing retrovirus and transplanted into lethally irradiated wild-type mice. Those who received HPCs with knocked-down *Kmt2d* expression in addition to increased *Bcl2* expression exhibited early onset of lymphoma, splenomegaly, and histopathological evidence of high-grade FL. A second mouse model, in which *Kmt2d* was knocked out completely, resulted in 58% of them becoming diseased compared to 0% in wild-type controls. The affected mice developed a lymphoma consisting of atypical pre-GC B-cells that had not undergone SHM or CSR. While the disease was not quite comparable to human lymphomas (e.g., FL and DLBCL), the results do support a role for KMT2D as a tumor suppressor. The third mouse model was designed to overexpress *Aicda* in addition to having a complete deletion of *Kmt2d*. Recall from earlier that *Aicda* encodes AID, which is essential for both the GC reaction and GC-derived lymphomas. All mice (7/7; 100%) developed lymphoma, whereas all of the mice overexpressing *Aicda* without *Kmt2d* deletion remained lymphoma-free (0/14; 0%)^99^. The onset of disease in affected mice was even earlier than in mice with *Kmt2d* deletion alone, and tumors displayed greater aggression and wider dissemination within the spleen and other organs. Evidence of SHM, CSR, and a plasmacytic phenotype were also present, none of which were observed in mice with *Kmt2d* deletion only. Experiments studying the effects of *Kmt2d* deletion on B-cell development produced results similar to those of Zhang et al.^98^. After SRBC immunization, there was a significant decrease in follicular B-cells and significant increases in transitional B-cells (also observed before immunization) and GC B-cells in the spleen. Using the same HPC transplant model described above, recipients of HPCs with knocked-down *Kmt2d* expression also exhibited prolonged GC formation in the spleen after SRBC immunization compared to wild-type mice. Furthermore, in vivo and in vitro experiments showed evidence of reduced CSR from IgM to IgG1 after immunization/stimulation in the B-cells of *Kmt2d*-knockout mice. These results differ from those of Zhang et al., which found an increase in IgG1+ B-cells but an approximately tenfold decrease in antigen affinity (though different antigens were used)^98^. Gene expression studies of *KMT2D*-mutant FL in humans and lymphoma in *Kmt2D*-deficient mice revealed a strong overlap between their sets of affected genes (compared to wild-type), especially those that are downregulated^99^. Notably, this overlap included genes involved in immune signaling (e.g., IL and TNF) and plasma cell differentiation. ChIP-seq experiments were also performed in murine *Kmt2d*-deficient lymphomas and human *KMT2D*-mutant lymphoma cell lines (compared to wild-type) in order to map changes in H3K4me1/2 distribution and associated changes in gene expression. In both mouse and human lymphomas, global levels of H3K4me1/2 did not decrease, but losses at specific loci were detected, especially at enhancers. In mice, some of the genes most affected by reduced H3K4me1/2 at promoters and enhancers were those that are induced by immune signaling (e.g., IL, TNF, NF-kB, and CD40); enhancer-specific H3K4me1/2 loss also overlapped with a variety of downregulated tumor suppressor genes. In human cells, genes that lost H3K4me1/2 and are known KMT2D binding targets were also heavily enriched for immune signaling pathways (e.g., IL, NF-kB, CD40, and IRF4). Lastly, multiple KMT2D target genes were studied in order to trace the direct connections between KMT2D mutation/loss and the functional effects that occur downstream. Many genes, such as tumor necrosis factor alpha-induced protein 3 (*TNFAIP3*), require BCR and CD40 signals for expression in B-cells. *TNFAIP3* encodes a protein (A20) that has been shown to negatively regulate NF-kB activity and promote apoptosis in non-Hodgkin lymphomas^100^. In human DLBCL cell lines with knocked-down *KMT2D* expression, the level of H3K4me1/2 at the enhancer of *TNFAIP3* (significantly) and its expression (not significantly) both decreased. Moreover, induction of *TNFAIP3* expression and cell apoptosis by BCR and CD40 signaling were both significantly decreased; these same results were observed in a comparison of *KMT2D*-mutant and *KMT2D*-wild-type human lymphoma cell lines, as was the absence of any significant effect on cell proliferation^99^. Thus, similar to the results of *CREBBP* inactivation that were discussed earlier (see section titled “The epigenetic switch at enhancers”), *KMT2D* loss/inactivation appears to make B-cells less responsive to CD40 signaling and less able to switch on genes that are required for important cell fate decisions.

Overall, the results of these functional studies suggest a multifaceted role for the loss/inactivation of *KMT2D* in the progression of lymphoma, especially in FL and DLBCL. The effects of its loss include increased proliferation, resistance to BCR and CD40 signaling, an expansion of GC B-cells at the expense of follicular B-cells, increased and prolonged formation of GCs, greater aggression, wider dissemination, and an increased capacity for lymphomagenesis when cooperating with other oncogenes. Finally, as an interesting side-note, although KTM2D is not directly involved in the acetylation or deacetylation of H3K27Ac, experiments in mouse embryonic stem cells and preadipocytes have shown that KMT2D (i.e., the protein itself, not H3K4 methylation) is required for CREBBP/P300 to bind and activate enhancers that regulate the expression of genes involved in terminal differentiation and cell identity^101–103^. Without jumping to conclusions, it would be interesting to know if mutations that result in decreased or lost KMT2D expression in GC B-cells also result in decreased CREBBP/P300 recruitment to and activation of enhancers in GC B-cells and/or lymphoma.

### Connections between epigenetic dysregulation and relapse in DLBCL

It is clear that mutations in histone-modifying enzymes like KMT2D, CREBBP, P300, and EZH2 are some of the most common in DLBCL and that they contribute directly to its molecular pathogenesis. The same can be said for FL, which can transform into and shares many similarities with DLBCL. There is a growing body of evidence suggesting that, in many instances, these are driver mutations that occur early on and help create an environment that is more conducive to oncogenesis. Moreover, these mutations are very commonly present in both early tumor cells and those that are selected during relapse or transformation from FL to DLBCL, thus indicating strong evolutionary fitness^104–108^.

**Fig. 6:**
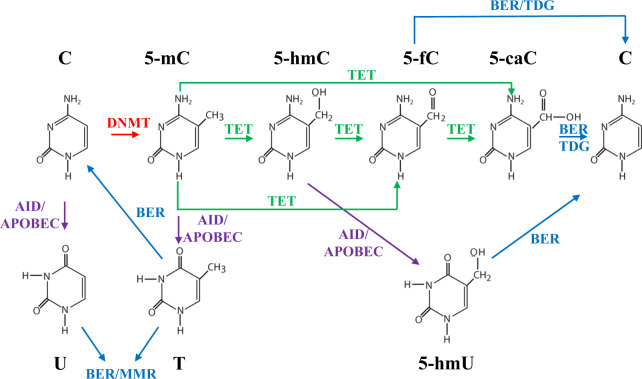
DNA repair and de-methylation pathways. Cytosine (C) methylation to 5-methyl cytosine (5-mC) is mediated by DNA-methyl transferases (DNMT) through direct transfer of CH3. De-methylation is a far more complex process, involving ten-eleven translocation (TET) enzymes, base excision repair (BER), and thymine DNA glycolase (TDG). Transition from C to uracil (U) or thymine (T) can also be involved in this complex process, through using activation-induced cytidine deaminase (AID) and apolipoprotein B mRNA-editing enzyme catalytic (APOBEC), and mismatch repair (MMR) (adapted from Bhutani et al.^80^ and Dominguez and Shaknovich^120^). 5-hmC: 5-hydroxy methyl cytosine; 5-fC: 5-formyl cytosine; 5-caC: 5-carboxyl cytosine; 5-hmU: 5-hydroxy methyl uridine.

For example, Jiang et al. compared matched pairs of tumors taken from 14 DLBCL patients, both at diagnosis and at relapse subsequent to R-CHOP therapy^107^. After analyzing the clonal heterogeneity of each sample and comparing each diagnostic tumor to its corresponding relapse tumor(s), two major patterns of clonal evolution were identified. The first was an “early-divergent” pattern in which the diagnosis and relapse clones were derived from a common precursor but diverged early on in development and occupied separate branches of the phylogenetic tree. The second was a “late-divergent” pattern in which the diagnosis and relapse clones occupied the same branch of the phylogenetic tree and retained a high degree of similarity. On average, early-divergent tumors had significantly greater entropy (i.e., clonal heterogeneity) at diagnosis than did late-divergent tumors. Furthermore, while the diagnosis subclones of early-divergent tumors were almost non-existent in relapse tumors, the diagnosis subclones of late-divergent tumors largely maintained their presence in relapse tumors. Exome sequencing was also performed on half of the matched pairs of tumors, including 3/6 from the early-divergent group. From an epigenetic perspective, the results from early-divergent tumors were particularly interesting, as all three pairs possessed mutations in histone-modifying enzymes in both the diagnosis and relapse tumors. Pair 1 had mutations in both *KMT2D* and *SETDB1* (SET domain bifurcated histone lysine methyltransferase 1), pair 2 had a mutation in *KMT2D* as well, and pair 9 had a mutation in *EP300*. Additionally, some of the relapse tumors contained additional chromatin-modifying/associated proteins that were not present at diagnosis. For pair 1, it was a mutation in *TET2* (tet methylcytosine deoxygenase 2), pair 2 had a mutation in *EP300*, and pair 9 added a mutation in *BRD4* (bromodomain-containing protein 4). TET2, along with TET1 and TET3, comprise the family of ten-eleven translocation (TET) that directs the convoluted process of cytosine demethylation. They catalyze the successive conversion of 5-methylcytosine (5mC) to 5-hydroxymethylcytosine (5hmC), 5-formylcytosine (5fC), and 5-carboxylcytosine (5caC), the latter two of which can trigger DNA repair and replacement with unmethylated cytosine^109^. Like AID, TET2 initiates a biological process that leads to reduced DNA methylation (Fig. 6) and an altered epigenome (see section titled “Endogenous sources of mutation and their signatures”). As a result of *TET2* loss, hematopoietic cells and acute myeloid leukemia (AML) cells displayed selective hypermethylation and inactivation of enhancers in one study^110^. Additionally, loss of *TET2* in GC B-cells was recently shown to mirror many of the mechanistic and functional effects observed in *CREBBP*-mutant DLBCL, including enhancer inactivation and inhibition of GC exit and plasma cell differentiation^111^ (Fig. 5).

BRD4 is an epigenetic “reader” that binds to acetylated histones and serves as a general transcription cofactor and partners with many transcription factors that promote gene expression. It is heavily involved in the organization and activation of super-enhancers, especially those that regulate the expression of genes that maintain cell identity. Interestingly, BRD4 is also involved in DNA repair and is required to complete CSR in B-cells after the introduction of DNA-DSBs by AID^112^. Additionally, one of the four late-divergent matched pairs of tumors (pair 8) also gained a mutation in a histone-modifying enzyme (EZH2) that was present only at relapse, not at diagnosis. Specifically, it was the Y641 EZH2 mutation, which disproportionately increases the deposition of H3K27me3 at the expense of H3K27me1/2, as reviewed earlier (see section titled “Frequent mutations in genes related to chromatin and epigenetics”). These findings, as well as those observed in FL^104^, led Jiang et al. to propose a model of DLBCL development and relapse in which mutations in epigenetic modifiers can serve as (1) early “driver” mutations that disrupt the epigenome in a way that favors lymphomagenesis and/or (2) “facilitator” mutations that occur later in development and introduce characteristics that favor relapse^107^. It was mentioned previously that *EZH2*, *CREBBP*, *EP300*, and *KMT2D* mutations in DLBCL predominantly have a signature of spontaneous cytosine deamination (C > T) that is associated with aging^16^. (See section titled “Frequent mutations in genes related to chromatin and epigenetics.”) Though it would need be shown definitively, this observation may further support the idea that mutations in histone-modifying enzymes can occur early on in the process (as a byproduct of aging; not sufficient for lymphomagenesis) and “set the stage” for lymphomagenesis later on.

**Fig. 7:**
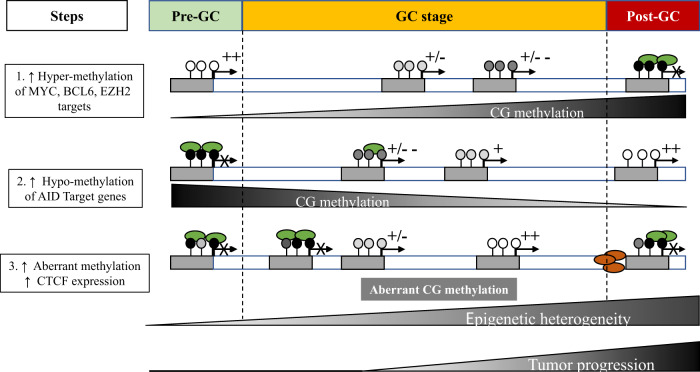
Activation-induced cytidine deaminase (AID), DNA methylation and epigenetic heterogeneity in DLBCL and other types of lymphoma. Activation of naïve B-cells (NBC) transitioning to normal germinal center B-cells (NGBC). Changes in DNA methylation patterns during differentiation (GC reaction) involve AID (see Fig. 6), which strongly contributes to creating epigenetic heterogeneity ultimately leading to disease progression and potentially aggressivity. Gray rectangle: promoter region; broken arrow; transcription start site (TSS); open circles: CG hypo-methylation; closed circles: CG hyper-methylation; gray circles: intermediate CG methylation state: 5-hmC, 5-fC, 5-caC, brown oval: CTCF, green oval: methyl-DNA binding proteins; ++: high expression; +: medium expression; +/−: Low expression: +/− −: very low expression; X: repressed.

This review of DLBCL epigenetics has focused primarily on histone-modifying enzymes because of their high mutation rates and their relevance to GC B-cell physiology and pathology. However, it is important to emphasize the role that DNA methylation plays in DLBCL^84,113–121^. Specifically, Pan et al. found that higher levels of DNA methylation heterogeneity in DLBCL tumors at the time of diagnosis could predict relapse subsequent to R-CHOP therapy^116^. Relapse was also accompanied by a decrease in intra-tumor DNA methylation heterogeneity, suggesting clonal selection. Furthermore, relapse was not correlated with clonal genetic heterogeneity, at least with regard to SHM patterns at VDJ sequences^116^. Additionally, Teater et al. recently published results from in vivo experiments in mice showing that AID itself is a driver of DNA methylation heterogeneity^118^ (Fig. 7). This implicates AID not only in the most essential steps of affinity maturation in normal GC B-cells, but also in the production of genetic and epigenetic heterogeneity during lymphomagenesis. The results of these studies make sense in light of what is known about AID’s involvement in DNA demethylation and epigenetic reprogramming. (See section titled “Endogenous sources of mutation and their signatures.”)

### Viewing DLBCL as a disease of epigenetic dysregulation

**Fig. 8:**
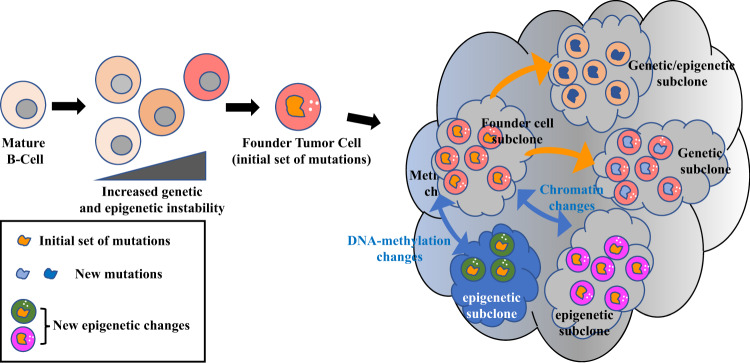
Epigenetic heterogeneity promotes the acquisition of aggressive traits in cancer. Classically, cancer has been thought of as a disease that is primarily genetic in nature. However, it is now known that epigenetic dysregulation in cancer can also be functionally and clinically relevant. One clear example is its contribution to tumor heterogeneity. When the epigenome is disrupted, either independently of genetic mutations (e.g., AID-related DNA hypomethylation) or as a result of them (e.g., in histone-modifying enzymes), tumor cells can start to evolve based on selection for favorable epigenetic states. This can lead to the production of tumor subclones that are genetically identical but, in reality, are expressing different combinations of genes and/or have altered the level at which certain genes are expressed. In addition to producing more aggressive characteristics, increased tumor heterogeneity decreases the likelihood that any one treatment will be able to kill every subclone, which can lead to chemoresistance and relapse (Based on work by Easwaran et al.^126^).

When attempting to synthesize all of these disparate findings regarding the epigenetic dysregulation of DLBCL, it is hard not to notice parallels with the epigenetic progenitor model of cancer proposed by Feinberg et al. in 2006 (refs. ^121–124^). Their model proposes that cancer really begins as a disruption of the epigenome in the stem/progenitor cells of a normal tissue prior to explicit oncogenesis. This produces a polyclonal population of cells that are epigenetically perturbed and begin gradually drifting toward oncogenesis. Epigenetic disruption could be caused by “tumor-progenitor genes” that “mediate epigenetic expansion of progenitor cells… and increase their cancer proneness, …” possibly by prioritizing stemness over differentiation^122^. Feinberg et al. specifically propose *AICDA* as a candidate tumor-progenitor gene because it acts on DNA directly, causes both genetic and epigenetic changes, and has been linked to B-cell lymphomagenesis. (See sections titled “Endogenous sources of mutation and their signatures” and “Connections between epigenetic dysregulation and relapse in DLBCL.”) They also point toward genes whose products directly modify chromatin, such as *EZH2*. Eventually, chronic epigenetic disruption leads to an initiating genetic mutation that formally drives oncogenesis, followed by further genetic and epigenetic aberrations. From then on, the cancer begins to increasingly emphasize the acquisition of genetic and epigenetic plasticity^125^, or “adaptability,” in order to more readily adapt to any conditions that it may encounter. For example, epigenetic instability is caused by changes in the expression of genes whose products modify chromatin, like EZH2. One could plausibly argue that changes in the activity of chromatin-modifying enzymes, including EZH2, CREBBP, P300 and KMT2D, may also lead to epigenetic instability^123,124^. Increased plasticity, in turn, fuels tumor heterogeneity and allows cancer cells to experiment with a diverse array of phenotypes and more aggressive characteristics (Fig. 8). This increases the entropy of the system and decreases the probability that any one therapy will kill every single subclone, which can lead to chemoresistance^121,126^. Once again, this sequence of events sounds quite similar to that which occurs during the development of DLBCL, a cancer notorious for both its heterogeneity and its resistance to treatment.

Additionally, it is evident that DLBCL is closely associated with aging. As was previously mentioned, the median age of diagnosis is 66 years old, and 66.3% of DLBCL cases occur between the ages of 55 and 84 (SEER 3, https://seer.cancer.gov/statfacts/html/dlbcl.html). Furthermore, ~80% of genetic mutations in DLBCL have a signature that is associated with aging^16^. It should be noted that the aging process itself is known to involve extensive epigenetic reprogramming. This includes such events as the loss of heterochromatin, changes in the levels of certain histone variants, alterations in the levels and distributions of histone PTMs and DNA methylation, and differential expression of noncoding RNAs^127^. It seems quite likely that the aging process contributes to the development of DLBCL in some patients, perhaps acting as an accelerant with regard to large-scale epigenetic reprogramming.

For all of these reasons, an enormous amount of both basic-science and clinical research has been devoted to finding alternative strategies for the treatment of DLBCL besides the standard R-CHOP chemotherapy regimen, especially those that target the epigenome^128–132^. Because epigenetic modifications are reversible, the ideas of reprogramming oncogenic epigenetic changes in DLBCL^133^ and preventing the transformation of precancerous B-cells^123^ are very appealing (This is the logic behind our on-going investigation into the effects of diet (i.e., ω-3 fatty acids) on the epigenome in DLBCL.). Theoretically, it stands to reason that “resetting” the epigenome of DLBCL cells could also potentially reduce tumor heterogeneity by eliminating subclones that are genetically identical but epigenetically distinct. Furthermore, if the drug doses were low enough to alter the epigenome and reduce tumor heterogeneity without killing cells, then perhaps it would be possible to shape the cancer’s characteristics and overall “trajectory” without promoting natural selection and the development of resistance. This would not eliminate the tumor mass itself, but if used rationally, perhaps some of its more aggressive characteristics and/or propensity for relapse could be reduced^133^. Much more research is needed to develop therapies for DLBCL that address its immense complexity in a way that R-CHOP chemotherapy simply does not.

## Supplementary information

Online References (SEER and UpToDate)
