# Supplementary material for: Genetic and epigenetic determinants of diffuse large B-cell lymphoma
Source: Blood Cancer J. 2020 Dec 4;10(12):123. doi: 10.1038/s41408-020-00389-w (PMC7718920; doi:10.1038/s41408-020-00389-w)
Supplement: Supplementary file 1 — Online References (SEER and UpToDate) [file 41408_2020_389_MOESM1_ESM.docx]

**Online References (in order of appearance)**

SEER 1: Cancer Stat Facts: Cancer of Any Site <https://seer.cancer.gov/statfacts/html/all.html>

SEER 2: Cancer Stat Facts: Non-Hodgkin Lymphoma <https://seer.cancer.gov/statfacts/html/nhl.html>

UpToDate 1: Epidemiology, clinical manifestations, pathologic features, and diagnosis of diffuse large B cell lymphoma <https://www.uptodate.com/contents/epidemiology-clinical-manifestations-pathologic-features-and-diagnosis-of-diffuse-large-b-cell-lymphoma?source=bookmarks_widget>

SEER 3: Cancer Stat Facts: NHL – Diffuse Large B-cell Lymphoma (DLBCL) <https://seer.cancer.gov/statfacts/html/dlbcl.html>

UpToDate 2.) Evaluation, staging, and response assessment of non-Hodgkin lymphoma <https://www.uptodate.com/contents/evaluation-staging-and-response-assessment-of-non-hodgkin-lymphoma?source=bookmarks_widget>

UpToDate 3.) Clinical presentation and diagnosis of non-Hodgkin lymphoma <https://www.uptodate.com/contents/clinical-presentation-and-diagnosis-of-non-hodgkin-lymphoma?source=bookmarks_widget>

UpToDate 4.) Prognosis of diffuse large B cell lymphoma <https://www.uptodate.com/contents/prognosis-of-diffuse-large-b-cell-lymphoma?source=bookmarks_widget>

UpToDate 5.) Initial treatment of advanced stage diffuse large B cell lymphoma <https://www.uptodate.com/contents/initial-treatment-of-advanced-stage-diffuse-large-b-cell-lymphoma?source=bookmarks_widget>

UpToDate 6.) Rituximab (intravenous) including biosimilars of rituximab: Drug information <https://www.uptodate.com/contents/rituximab-intravenous-including-biosimilars-of-rituximab-drug-information?source=bookmarks_widget>

UpToDate 7.) Cyclophosphamide: Drug information <https://www.uptodate.com/contents/cyclophosphamide-drug-information?source=bookmarks_widget>

UpToDate 8.) Doxorubicin (conventional): Drug information <https://www.uptodate.com/contents/doxorubicin-conventional-drug-information?source=bookmarks_widget>

UpToDate 9.) Vincristine (conventional): Drug information <https://www.uptodate.com/contents/vincristine-conventional-drug-information?source=bookmarks_widget>

UpToDate 10.) Prednisone: Drug information <https://www.uptodate.com/contents/prednisone-drug-information?source=bookmarks_widget>

UpToDate 11.) Initial treatment of limited stage diffuse large B cell lymphoma <https://www.uptodate.com/contents/initial-treatment-of-limited-stage-diffuse-large-b-cell-lymphoma?source=bookmarks_widget>

UpToDate 12.) Pathobiology of diffuse large B cell lymphoma and primary mediastinal large B cell lymphoma <https://www.uptodate.com/contents/pathobiology-of-diffuse-large-b-cell-lymphoma-and-primary-mediastinal-large-b-cell-lymphoma?source=bookmarks_widget>

SEER 3: Cancer Stat Facts: NHL – Diffuse Large B-cell Lymphoma (DLBCL) <https://seer.cancer.gov/statfacts/html/dlbcl.html>
